# Novel elastomeric spiropyran-doped poly(dimethylsiloxane) optical waveguide for UV sensing

**DOI:** 10.1007/s12200-024-00124-4

**Published:** 2024-07-15

**Authors:** Camila Aparecida Zimmermann, Koffi Novignon Amouzou, Dipankar Sengupta, Aashutosh Kumar, Nicole Raymonde Demarquette, Bora Ung

**Affiliations:** 1 Department of Electrical Engineering, École de Technologie Supérieure, Montreal, QC H3C 1K3 Canada; 2 Department of Mechanical Engineering, École de Technologie Supérieure, Montreal, QC H3C 1K3 Canada

**Keywords:** Spiropyrans, PDMS, Photochromism, Polymer optical waveguides, UV detection

## Abstract

Novel poly(dimethylsiloxane) (PDMS) doped with two different spiropyran derivatives (SP) were investigated as potential candidates for the preparation of elastomeric waveguides with UV-dependent optical properties. First, free-standing films were prepared and evaluated with respect to their photochromic response to UV irradiation. Kinetics, reversibility as well as photofatigue and refractive index of the SP-doped PDMS samples were assessed. Second, SP-doped PDMS waveguides were fabricated and tested as UV sensors by monitoring changes in the transmitted optical power of a visible laser (633 nm). UV sensing was successfully demonstrated by doping PDMS using one spiropyran derivative whose propagation loss was measured as 1.04 dB/cm at 633 nm, and sensitivity estimated at 115% change in transmitted optical power per unit change in UV dose. The decay and recovery time constants were measured at 42 and 107 s, respectively, with an average UV saturation dose of 0.4 J/cm^2^. The prepared waveguides exhibited a reversible and consistent response even under bending. The sensor parameters can be tailored by varying the waveguide length up to 21 cm, and are affected by white light and temperatures up to 70 ℃. This work is relevant to elastomeric optics, smart optical materials, and polymer optical waveguide sensors.

**Graphical Abstract:**

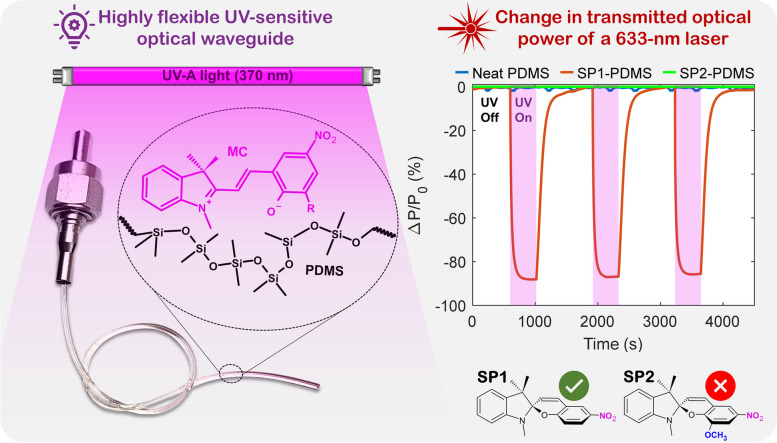

**Supplementary Information:**

The online version contains supplementary material available at 10.1007/s12200-024-00124-4.

## Introduction

Ultraviolet (UV) light is an important form of electromagnetic radiation. It composes around 5% of the solar terrestrial radiation, of which 95% is UVA (315–400 nm) at midday sun [1, 2]. On the one hand, UV light is paramount for human health [2–4], is part of wastewater treatments [5, 6], sterilization protocols [7, 8], additive manufacturing [9], phototherapy [10, 11], photopolymerization [12–14], photolithography, spectroscopy, and plays a role in plant growth [15, 16]. On the other hand, UV radiation is considered carcinogenic over its entire wavelength range (100–400 nm) with a larger effect at the shorter end [1, 3]. It is associated with vision impairment [4], premature skin aging [2], and is the main cause of polymer photodegradation [7, 17]. The beneficial or harmful effects are dependent, among other variables, on the delivered UV dose [2]. Thus, monitoring UV radiation is critical to safely harnessing its full potential.

Typical UV photodetectors rely on wide bandgap semiconductors, such as gallium nitrides [Al, In(GaN)], gallium oxide (Ga_2_O_3_), silicon carbide (SiC), diamond, silicon (Si), zinc oxide (ZnO) [18, 19], tin oxide (SnO_2_) [20], and perovskites [21]. Detection is based on the photoelectric effect where UV radiation is converted into an electrical signal [22]. These inorganic stiff materials require specialized fabrication facilities and are unsuitable for emerging applications requiring dynamic mechanical regimes, high strain, and shape-conforming contour coverage over large areas. In this regard, flexible polymers are a potential material platform for developing a novel generation of flexible UV sensors, as evidenced by recent reviews on the subject [20, 22–26].

Among the various UV-detecting modalities, optical sensors present several advantages over photoelectric ones, including immunity to electromagnetic interference and corrosion, as well as no constraints related to the integrity of a conductive path. Optical UV sensing is typically achieved with the use of materials that change optical properties, e.g., color, luminescence, and transparency upon UV exposure [22, 26]. For instance, polysulfone films, which have been considered the gold standard for UV dosing, irreversibly photodegrade, altering their color. The total color change can be later converted to cumulative UV dose using calibration curves and a spectrophotometer [23, 26]. Photochromic dyes and polymers have piqued particular interest of researchers in the development of flexible sensors in the form of films and textiles. UV detection is accomplished by visual inspection with naked eyes and colorimetric assessment with spectrophotometers and smartphones [27–38]. Being a color a psychophysical evaluation, it depends on the ambient light (illuminant), the angle of observation, the visual acuity of the observer, and the properties of the observed item such as texture. Thus, inconsistent assessment may be an issue during color inspection with bare eyes or even with the help of a smartphone [34]. In addition, access to a spectrophotometer is nontrivial for consumer applications. In this sense, dye-doped optical waveguides are considered a promising and straightforward approach to monitoring UV radiation dose by monitoring changes in transmitted optical power through commonly available LED/laser source and photodiode, guarantying its integrability to optoelectronics [39–42]. The waveguide-based configuration is advantageous compared to films and textiles by providing a more reliable, quantifiable and simpler means for UV detection that do not rely on visual inspection or spectral analysis, with improved sensitivity. Ock et al., for example, prepared a planar waveguide composed of poly(methyl methacrylate) (PMMA) doped with 1 wt% spirooxazine, whose sensitivity improved by 64 times compared to the transmittance method. The authors also demonstrated that the sensitivity could be modulated by the dye concentration [40]. Moreover, Chen and Wang proposed a highly responsive photochromic optical waveguide based on a coil of ZBLAN glass doped with spirooxazines and naphthopyrans [39]. Considering that the beneficial or harmful effects of UV exposure on human health are dose-dependent [1–4], a highly flexible UV sensing waveguide also offers a potential platform for integration with wearable devices, providing a more comfortable and convenient monitoring alternative to traditional stiff semiconductor UV photodetectors.

**Fig. 1:**
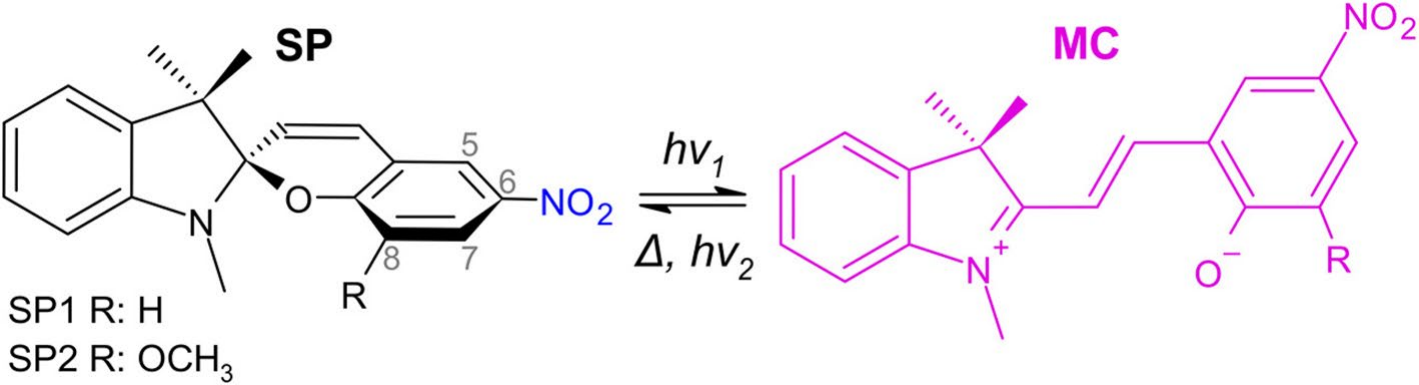
Chemical structure representation of spiropyrans SP1 and SP2 photochromic dyes and their conversion to the colorful (purplish) merocyanine form

Nitro-substituted spiropyrans (SPs) are well-known photochromic dyes which undergo a ring-opening reaction forming the colorful merocyanine (MC) triggered by UV radiation [43–45], as illustrated in Fig. 1. They have a number of appealing characteristics, including fast and reversible response, no need for thermal treatment for recovery, commercial availability, compatibility with a variety of materials and common organic solvents, tunable chemistry, and contrasting optical properties between isomers. In this work, we explore the spiropyrans’ potential as a doping agent for poly(dimethylsiloxane) (PDMS) in order to prepare UV sensitive PDMS optical waveguides. PDMS was chosen as the polymer matrix because it is highly transparent in the UV-Vis range, it is flexible, stretchable, biocompatible, possesses good thermal and chemical resistance and can be processed at room temperature. Additionally, PDMS is generally more resistant to UV photooxidation than other organic polymers, thanks to the higher energy required to break its Si–O bonds [46, 47]. To the best of our knowledge, this is the first time that a spiropyran-doped elastomer is employed as a flexible optical waveguide for UV detection.

First, we described the procedure to fabricate free-standing films and air-clad waveguides from PDMS doped with two different spiropyrans. Subsequently, the free-standing films were characterized under UV-A irradiation in order to better understand the materials’ behavior and draw some conclusions. Kinetics, photofatigue, and the refractive index were examined. Finally, the response of air-clad SP-doped PDMS waveguides was investigated via changes in the optical transmitted power of a red He-Ne laser. The waveguides were subjected to different test conditions, including temperature increases and bending. Based on the testing results, we demonstrated the feasibility of using SP-doped PDMS as a novel material for optical UV sensing.

## Materials and methods

### Chemicals and sample preparation

Two spiropyrans possessing a nitro group at the 6-position of the benzopyran ring (highlighted in blue in Fig. 1) were mixed with PDMS using a solvent-based approach. Their chemical structure representations are shown in Fig. 1: 1′,3′-Dihydro-1′,3′,3′-trimethyl-6-nitrospiro[2H-1-benzopyran-2,2′-(2H)-indole] (**SP1**, purity 98%) and 1′,3′-Dihydro-8-methoxy-1′,3′,3′-trimethyl-6-nitrospiro[2H-1-benzopyran-2,2′-(2H)-indole] (**SP2**, purity 97%), both in powder form and procured from Sigma-Aldrich (USA). In contrast to the SP1 chemical structure, the SP2 compound presents a methoxy group (–OCH_3_) at the 8-position of the benzopyran moiety that antagonizes the electron-withdrawing effect of the nitro group (‒NO_2_) at the 6-position and is believed to enhance the photofatigue resistance [45]. The presence of another substituent also results in changes in the UV-Vis spectrum and kinetic parameters [45, 48, 49].

PDMS Sylgard 184 (Dow Corning, USA) was prepared at a 10:1 base-to-curing agent ratio following the manufacturer’s technical data sheet [50]. SP concentration was fixed at 0.05 wt% on a dry basis because increasing the content to 0.1 wt% resulted in undesired crystallization. First, 1.5 mg/mL of SP solution was prepared by dissolving 2.75 mg of spiropyran in 2.75 g of chloroform (Sigma Aldrich, ≥ 99.8%). After stirring for 10 min using a magnetic stirrer, 5 g of PDMS base were added to the solution. The mixture was kept in an open beaker under a fume hood and stirred overnight (18 h) for solvent evaporation. During waveguide fabrication, it is important to keep the solvent concentration to a minimum as it is a health hazard, may compromise the mechanical properties [51], and cause shrinkage or warpage with its evaporation. Solvent evaporation was monitored by weight loss. By the end of the 18-h period, the mixtures had lost an average of 96 wt% of the initial solvent and reached a roughly constant weight, indicating that the majority of the solvent had evaporated. The PDMS curing agent (0.5 g) was added to the mixture and stirred for 10 min with a magnetic stirrer, then manually for another 2 min. The mixture was filtered with a 75-µm pore-size nylon filter to remove dust particles and most of the bubbles. Finally, the mixture was degassed and injected into a PTFE tube (high-temperature tube sleeving, Ø_i_ = 1 mm, 30 cm long, McMaster-Carr, USA). Before demolding, the waveguides were allowed to cure for 23 days at 22 ± 1 ℃ inside the tubes. The extraction was performed by cutting the tubes lengthwise. Neat PDMS samples were prepared following all the same fabrication steps, including the addition and evaporation of solvent, except for the addition of the dopants.

Moreover, free-standing films were prepared by the floating-on-water technique according to Kim et al. with some modifications [52]. The technique consists of carefully pouring PDMS into a floating frame suspended in a container filled with room-temperature water. After 48 h, all samples had been properly cured. After which, they were removed from the water bath, dried, and stored in the dark for a couple of weeks prior to testing. The films presented an average film thickness of 440 ± 160 µm, measured with a micrometer gauge.

### Characterization

The refractive index (RI) of the free-standing films (*n*) was measured after 209 days of curing using an Abbe refractometer (AR-2, Azzota LLC) operated with ambient light and a He-Ne laser in a dark room. A CCD camera was mounted on the eyepiece for the safe operation of the refractometer and to keep the angle of observation constant. A uniform illumination was obtained by passing the He-Ne laser beam through a frosted plastic sheet diffuser. The temperature at the end of each reading (*T*) was taken and used to calculate the RI at 20 ℃ (*n*^20^) according to Eq. (1). The results are given as an average of measurements performed on five specimens per composition.

1 $${n}^{20}={n}^{T}+0.00045\left(T-20\right).$$

After the initial RI reading, the samples were exposed one by one to UV-A light for 3 min using a hand-held lamp (37 W/m^2^ at 370 nm wavelength, F15T8/BL, 15 W, Hitachi Ltd., Japan) fixed at 15 cm from the samples inside a dark chamber. All samples were tested, including the neat PDMS, to check for possible RI changes induced by the irradiation. The RI measurements were taken immediately after removal from the UV chamber (~ 15 s).

The cutback technique was employed in measuring the propagation loss of neat PDMS and SP-doped PDMS waveguides when coupled to a non-polarized white light source (peak at 660 nm, HL-2000-HP, Ocean Optics Inc.) or a He-Ne laser (633 nm, 05-LHP, Melles Griot Inc.) while the transmitted optical power was measured with a photodiode (S120C, Thorlabs Inc.) and a power meter interface (PM100USB, Thorlabs Inc.). After each cut length, the waveguides were irradiated with UV-A light until the measured transmitted power reached stabilization. New measurements were taken after power intensity recovery in the dark in room conditions. The propagation loss was calculated as the slope of a linear regression in which total optical loss ($10{\text{log}}_{10}{(P}_{\text{in}}/{P}_{\text{out}})$, dB) was plotted as a function of the propagation length (cm). The output spectra of the waveguides, UV-A lamp, white light source, and LED white light lamp used in this work were collected using an optical fiber (QP600-1-SR, Ocean Optics Inc.) connected to a high-resolution spectrometer (HR4000CG, Ocean Optics Inc.). The spectra of the light sources are available in Supporting Information (SI), Fig. S1.

The photochromic response of the SP-doped PDMS mixtures was first evaluated on free-standing films. The tests consisted of exposing them to UV-A light (370 nm, 15 cm of distance). The exposure to UV-A light was carried out until the samples reached the photostationary state, i.e., when the maximum absorbance intensity became roughly constant (5 min). Absorbance measurements were carried out using a UV-Vis spectrophotometer (Cary 60, Agilent Inc.) from 190 to 1100 nm, with a step size of 5 nm. The sample distance from the beam was adjusted for a centralized tested area of 4 mm × 4 mm. The average time taken to move the samples between the UV chamber and the spectrophotometer sample compartment was 5 s during the coloring process. After that, the samples were kept in the dark inside the spectrophotometer except during the scanning in the visible range, with a duration of 5 s per scan. Results were at least triplicated and collected at 23 ± 1 ℃ and 15 ± 5% RH (relative humidity). A baseline in free space (without a sample) was collected and subtracted during data processing. The spectra of the neat PDMS samples were also recorded for reference. A total of 23 irradiation cycles were performed for photofatigue evaluation. Transmittance values were normalized between 0 and 1, where 0 corresponds to the minimum transmittance value reached at the first UV irradiation cycle for each sample, and 1 to the maximum transmittance collected before UV irradiation (baseline).

The UV sensing with 10-cm-long waveguides was subsequently investigated. The He-Ne laser (633 nm) was chosen as the light source because one of the spiropyrans (SP1) was found to be highly responsive at 633 nm, as detailed further below. SMA connectors were attached to the waveguide ends, resulting in a total of 5 cm exposed waveguide length. The setup was placed inside a black hardboard enclosure, in a dark room, to avoid interference from ambient light. Laser stabilization was ensured by turning it on for at least 1.5 h prior to testing. The UV-A lamp was fixed at a distance of 15 cm from the top of the waveguide under test. Room temperature and humidity measurements were taken with a USB temperature and humidity data logger (TSP01, Thorlabs Inc.). The temperature of the waveguides was monitored in real time with two external temperature probes. These probes were evenly spaced along the length and laterally less than 1 mm apart from the waveguide. All sets of experiments were carried out at 23.5 ± 1.0 ℃ and 58 ± 3% R.H. unless stated otherwise following the general procedure detailed above.

In the first set of experiments, the waveguides’ response to UV light was evaluated in the dark. Three exposure regimes were applied to each of the three waveguides per composition to test their reversibility under different UV durations corresponding to different UV doses:

- First cycle regime: 5 × [1 min UV-A (dark) + 5 min of recovery in the dark],
- Second cycle regime: 5 × [3 min UV-A (dark) + 20 min of recovery in the dark],
- Third cycle regime: 1 × [15 min UV-A (dark) + 40 min of recovery in the dark],

where 1, 3, and 15 min of UV-A light correspond to doses of 2.2, 6.7, and 33.6 kJ/m^2^, respectively. The UV doses were calculated by multiplying the UV irradiance by the UV exposure time.

The next sets of experiments were performed only with SP1-PDMS because the transmitted optical power of SP2-PDMS waveguides was near zero even before UV irradiation, owing to the large optical losses for that material, as detailed in Section 3.2. Results for neat PDMS waveguides were included for comparison purposes.

#### Length effect

By increasing the waveguide length, we can expect a larger effect of propagation loss at play, small fluctuations in composition along the length, and changes in sensing performance. To assess the UV sensing parameters of longer SP1-PDMS waveguides, samples measuring 15 and 19 cm in length were also tested, and the results were compared to 10-cm-long samples. For each length, the waveguides were exposed to 3 cycles of UV light, followed by recovery in the dark.

#### Bending effect

**Fig. 2:**
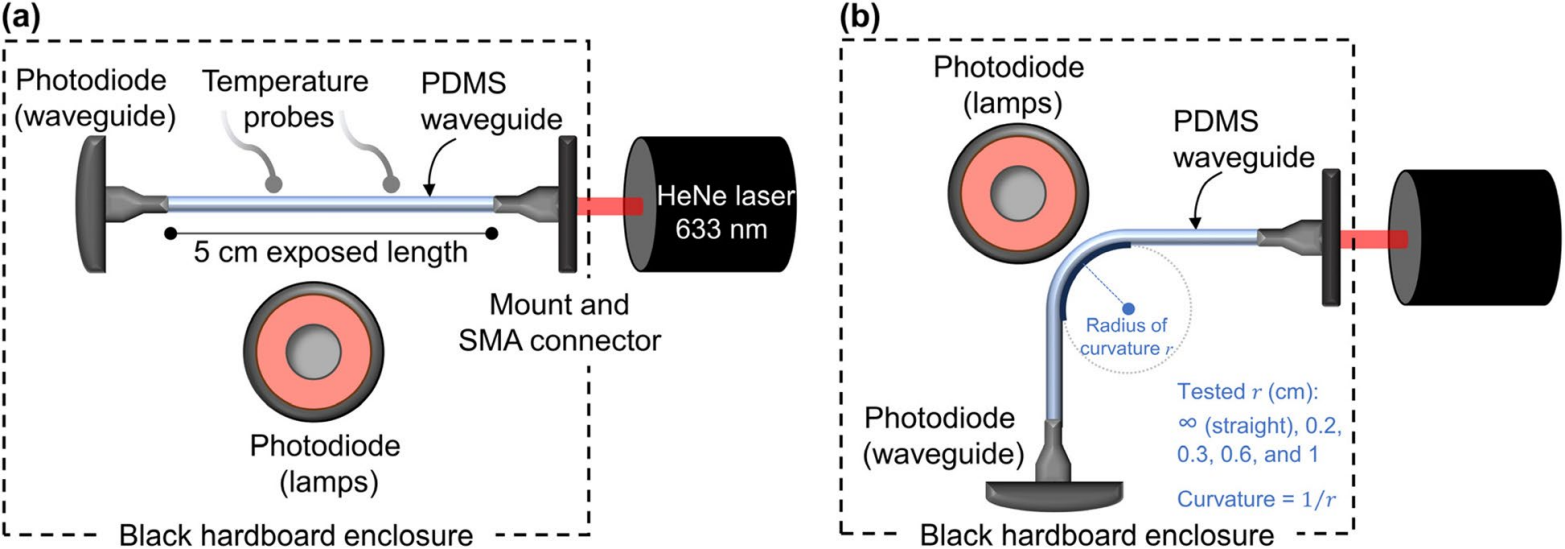
**a** Top view of the optical setup used to test the response to UV light in the dark, UV light combined with white light, temperature, and length effects of straight waveguides. **b** Optical setup for the evaluation of the bend-induced optical response

In practice, a flexible waveguide will experience mechanical strain while in service. One important type of perturbation concerns the bending of a waveguide, which combines tension and compression. To study this effect, the waveguides’ optical losses were evaluated at a 90° bending around metallic rods of varied sizes. The actual angles and diameters were measured from images of the waveguides assembled in the setup using ImageJ software. The average angle was 92 ± 2°, with average bend radii of 0.21 ± 0.01, 0.34 ± 0.04, 0.62 ± 0.04, and 1.01 ± 0.04 cm. Bend loss (${\alpha }_{\text{B}}$) was determined using the slope of the best-fit linear regression of total optical loss ($10{\text{log}}_{10}{(P}_{\text{straight}}/{P}_{\text{bent}})$, dB) versus curvature (cm^−1^). Curvature was calculated as the reciprocal of the radius of curvature in cm. Figure 2 shows a schematic of the experimental optical setups. One photodiode (S120C, Thorlabs Inc.) was used to measure the waveguide output power [photodiode (waveguide)], while another to monitor the irradiance of the UV-A lamp delivered to the samples [photodiode (lamps)].

#### White light effect

Given that white light irradiation promotes the conversion of colorful MC back to SP form, we investigated the competing effect of keeping the white light on during UV exposure. For that, a white light LED lamp was positioned 25 cm from the waveguides (18 W/m^2^ at 603 nm, PLYB1002D, 6 W, 2700 K daylight, Luminus Inc). The same three aforementioned exposure regimes were applied. This time, however, the white light remained on throughout the experiment.

#### Temperature effect

Another parameter that can have a significant impact on sensor performance is temperature, which affects not only reaction rates but also causes thermal expansion of PDMS. To evaluate the thermal effects on UV sensing and find the maximum working temperature, a hotplate (Cosori CO294-CW, Vesync Co.) was added to the setup. The waveguides were in direct contact with the hot plate for efficient heat transfer. Their temperature was adjusted in order to reach the target value as monitored by the TSP01 temperature probes after a stabilization period of 10 min. Since the waveguides are relatively small, we assume a uniform heat distribution over the whole waveguide volume. The experiment was performed under continuous white light exposure as detailed above. The UV light was turned on once the temperature had stabilized near the target value. The samples were tested at room temperature (24 ℃), and at approximately 30, 40, 50, 60, and 70 ℃. One cycle of UV-white light irradiation followed by recovery in white light was performed per temperature level and per waveguide. A total of three waveguides were tested per composition.

The temperature-induced optical loss *α*_*T*_ that followed the increase in temperature was estimated by the slope of the total optical loss in dB/cm, calculated according to Eq. (2), as a function of temperature:

2 $${\alpha }_{T}={\alpha }_{0}+\frac{10}{L}{\text{log}}_{10}\left(\frac{{P}_{i}}{{P}_{T}}\right),$$

where *α*_0_ is the propagation loss at 633 nm at room temperature (Table 3), *L* is the waveguide length, *P*_*i*_ is the initial transmitted optical power at 633 nm at room temperature, and *P*_*T*_ is the optical power at a temperature *T*.

The response (and recovery) is related to SP conversion into MC (and vice versa), as illustrated in Fig. 1. The generation of the absorptive MC species is observed by an exponential decay in transmittance in the visible range with UV-A (*hν*_1_) exposure time. The opposite happens in recovery, where transmittance grows exponentially. The latter is a thermally driven process that can be accelerated by heat (Δ), white or green light (*hν*_2_). The rate constants (*k*) related to each process were obtained by fitting the transmittance peak evolution with time according to Eq. (3):

3 $${{T}_{r}}^{\pm }={C}^{\pm }\pm {A}_{\pm }\text{exp}\left(-{k}_{\pm }t\right),$$

where *T*_*r*_ is the normalized transmittance (a.u.) at a given time *t*, *A* is the pre-exponential factor, and *C* is a constant.

The rate constants can be converted to decay and recovery time constants, *τ*_*d*_ and *τ*_*r*_, by taking their reciprocal. The time constant of a system’s response with exponential decay corresponds to the time when the response has decreased by 37%. Meanwhile, in a system with exponential growth, the time constant represents the amount of time required for the system response to reach approximately 63% of its final value. Experimental data fitting was performed using Matlab and Microsoft Excel.

## Results and discussion

### Preparation and characterization of free-standing films

**Fig. 3:**
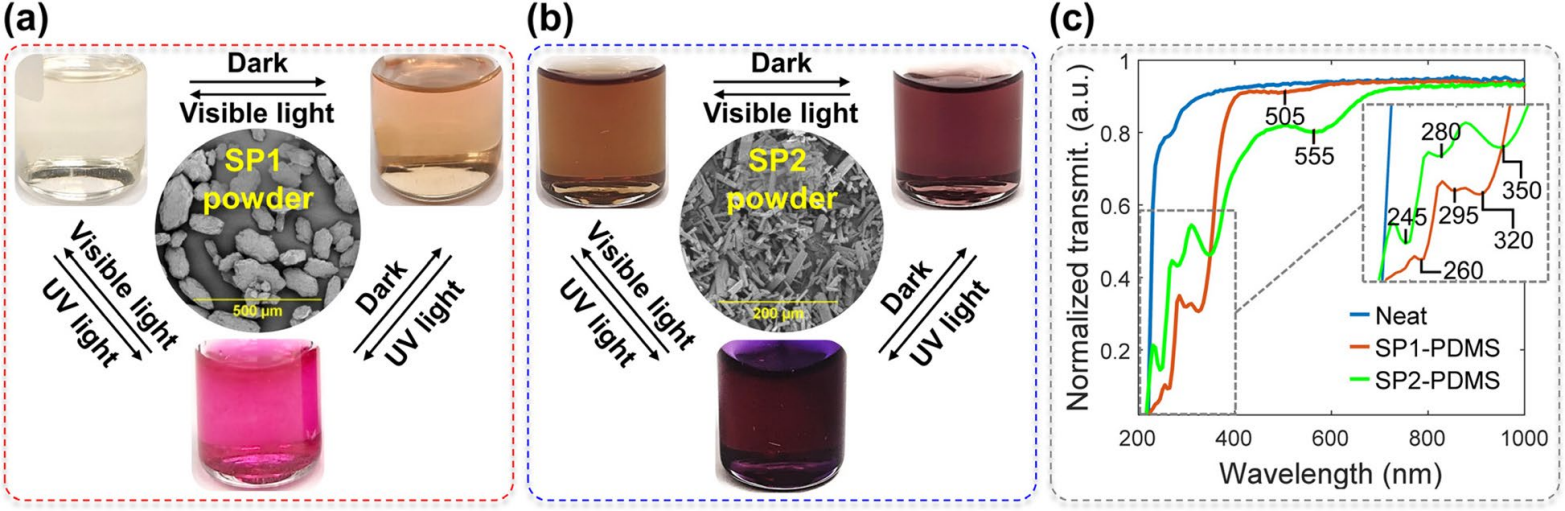
**a**, **b** Pictures of the SP-doped PDMS liquid mixtures after solvent evaporation under different conditions: exposure to visible light for 10 min, to UV light for 3 min, and 5 min in the dark. SEM images of the SP powders were obtained with a scanning electron microscope (S3600-N, Hitachi). The powders were deposited on carbon tape fixed on a stub and gold coated. **c** Normalized transmittance spectra of neat and SP-doped PDMS films collected after dark storage

After evaporating chloroform, the mixture of SP1 and PDMS (SP1-PDMS) yielded a pale-yellow, clear viscous liquid, as shown in Fig. 3a. When stored in the dark, this material appears pale orange, and when exposed to UV-A radiation, the mixture turns pink (Fig. 3a). These findings are consistent with the existing literature [53]. After solvent evaporation, the SP2-doped PDMS (SP2-PDMS) mixture showed a brownish-red coloration that intensified when left in the dark. A deep purple tint is observed after UV-A exposure (Fig. 3b). The cured free-standing films and waveguides produced from these mixtures retained the same color behavior. Figure 3c is a plot of the normalized transmittance of the different films collected after 24 h of dark storage.

As given in Fig. 3c, the neat PDMS film is highly transparent (> 87%) between 300 and 1000 nm, becoming completely opaque below 220 nm. The SP-doped films presented three absorption bands in the UV region, with the two bands below 300 nm ascribed to the π-π* transition of the carbon-carbon double bonds present in the spiropyran structure and another between 320 and 350 nm characteristic of the benzopyran structure [44]. These are important spectral characteristics to allow a full SP to MC conversion throughout the waveguide cross-section by external UV light sources with peak emission > 230 nm. Both SP-doped materials exhibited one absorption band in the visible range which is related to the colored appearance of the samples in the non-irradiated state stored in the dark. The SP1-PDMS films presented a broad absorption band of very weak intensity, centered at 505 nm (Fig. 3c). Meanwhile, SP2-PDMS films have a significantly larger absorption that extends further into the visible range with a peak at 555 nm, which matches the merocyanine peak evolution described later on. The presence of a colored state in the absence of ambient light (darkness) is typically referred to as negative photochromism (more details provided in the SI, item 3).

**Fig. 4:**
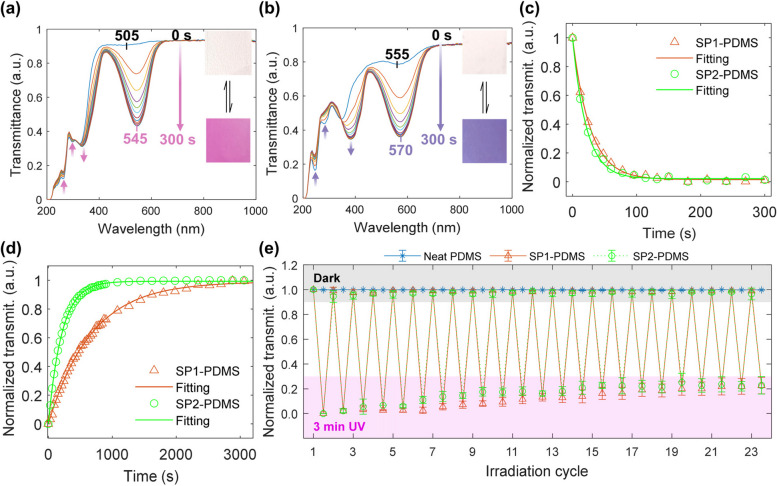
Representative transmittance spectra evolution of **a** an SP1-PDMS and **b** an SP2 PDMS films as a function of UV-A exposure time, from 0 to 300 s of accumulated irradiation. Inset images show the doped PDMS films before and at the end of the irradiation process. Representative fitting curves of transmittance **c** decay with UV irradiation and **d** recovery in the dark. **e** Variation in normalized transmittance as a function of irradiation-recovery cycles. Error bars show the sample standard deviation in independent measurements on three films per composition

Overall, SP1 and SP2 are well distributed and finely dispersed in the PDMS free-standing films, as confirmed by polarized optical microscopy micrographs (available in SI, Fig. S2). The transmittance spectra of the SP-doped PDMS films show significant changes as the UV irradiation progresses, as shown in Fig. 4a and b. These changes are indicated by arrows and are accompanied by a visible color change, as given in the insets of Fig. 4a and b. The normalized transmittance intensities of the MC peaks over time were used to calculate the time constants according to Eq. (3). Representative experimental data and fitted curves for both decay and recovery in transmittance are shown in Fig. 4c and d. The normalized transmittance values over the course of 23 UV-recovery cycles are presented in Fig. 4e.

As shown in Fig. 4a, the SP1-PDMS absorption band initially centered at 505 nm, shifts to approximately 545 nm with UV-A irradiation, resulting in a peak absorption shift of 40 nm. Similarly, the SP2-doped PDMS absorption band at 555 nm shifts to 570 nm. The observed spectral changes are the results of intermolecular interactions and structural changes involved in the isomerization reaction from the close and non-planar spiropyran form to the planar merocyanine form, illustrated in Fig. 1. The planar configuration and opening of the spiro ring decrease the energy required for electronic transition and increase the conjugation length (number of conjugated double bonds), giving rise to the MC peak observed in the visible [44]. This strong absorption also changes the color of the doped films, as shown in the insets of Fig. 4a and b. The MC peak shifts observed upon UV irradiation can be explained by an increase in the proportion of MC species along with changes in the average energy levels caused by the increased contribution of MC-PDMS/chloroform and MC-MC interactions, and their effect on charge distribution in the molecules [54]. Recovery curves exhibit the inverse evolution of the transmittance spectra shown in Fig. 4a and b. Above 700 nm, no relevant variation in transmittance was observed. For both spiropyrans, the typical MC absorption band is absent of shoulders that are otherwise indicative of MC aggregation [44, 48, 54]. Despite the low transition temperatures of PDMS, the dipole-dipole interactions (Fig. S3a) are strong enough to stabilize the MC form, thus preventing its aggregation.

With regards to the transmittance decay and recovery curves (Fig. 4c and d), all experimental data were well fitted via Eq. (3) with *R*^*2*^ > 0.99, and their respective time constants are presented in Table 1.

**Table 1:** Transmittance decay and recovery time constants and MC absorption peak for 4 irradiation cycles

Cycle	SP1-PDMS		SP2-PDMS	
	*τ*_*d*_ (s)	*τ*_*r*_ (s)	*τ*_*d*_ (s)	*τ*_*r*_ (s)
1	29 ± 3	556 ± 20	22 ± 2	201 ± 10
2	28 ± 2	626 ± 14	21 ± 1	207 ± 19
3	28 ± 1	543 ± 18	20 ± 1	183 ± 18
4	27 ± 2	662 ± 11	20 ± 2	200 ± 20

Based on the results presented in Table 1, SP1-doped PDMS takes a little longer than SP2-PDMS to reach the photostationary state upon UV irradiation. Meaning that it is able to measure a higher UV dose before saturation. The variations in *τ*_*d*_ as a function of the irradiation cycle for each material are within the standard deviations. Meanwhile, SP1-PDMS has a recovery period (to return to the initial transmittance level) that is almost three times longer than for SP2-PDMS. The slower SP1-PDMS recovery in polar medium corroborates with the smaller rate constant of ring closure reported by Berman et al. [49]. In both cases, *τ*_*r*_ increased and decreased from one cycle to another, being larger in the second and fourth cycles without a clear trend. As fading is a thermally driven process, we believe that small local temperature fluctuations could be the source of such variations, even though the registered average room temperature varied between 22.8 and 23.1 ℃ during the test. The differences in *τ*_*d*_ and *τ*_*r*_ are explained by the different photoswitching mechanisms involved in the ring-opening and closing processes [43].

Photofatigue is expected to occur due to photooxidation, reaction with solvent and impurities, and aggregation [45]. Systems where the spiropyran is not crosslinked to the polymer backbone (chemical doping), the spiropyran contains a nitro group (‒NO_2_), and the matrix allows for high molecular mobility (high free volume, low *T*_g_), are particularly prone to photofatigue [44, 45, 48]. Still, physical doping is preferred over chemical doping because it does not require specific functional groups and allows greater material versatility. A decrease in the maximum absorption intensity at the irradiated state, also observed as an increase in normalized transmittance with each new UV irradiation cycle, is a sign of photofatigue, as shown in Fig. 4e (lower shaded pink area). SP1-PDMS preserved more than 90% of the initial transmittance in the irradiated state for the first 10 cycles, whereas SP2-PDMS presented an average intensity retention below 90% already in the sixth cycle. The average retention at the end of the twenty-third cycle was approximately 77 ± 7% for both materials. By fitting the transmittance increase with irradiation cycles with a linear regression (see SI, Fig. S4), SP1- and SP2-PDMS normalized transmittances are expected to reach 50% after 50 and 39 irradiation cycles, respectively. The results obtained for the two different spiropyrans may be explained by the reaction rate sensitivity of the substituent groups to the polarity of the medium (PDMS and chloroform) and competing isomerization reaction mechanisms [48].

The refractive index (RI) of the free-standing films was measured with an Abbe refractometer operated in ambient light and at 633 nm (He-Ne laser). After the initial RI reading, the samples were exposed one by one to UV-A (370 nm) for 3 min, which is enough for full decay in transmittance (Fig. 4c, Table 1). The experimental results are summarized in Table 2.

**Table 2:** Refractive index at 20 ℃ of undoped and SP-doped PDMS films, before and after UV irradiation

Sample	RI^20^ in ambient light		RI^20^ at 633 nm	
	Before UV irradiation	After UV irradiation	Before UV irradiation	After UV irradiation
Neat PDMS	1.4136 ± 0.0002	1.4135 ± 0.0001	1.4136 ± 0.0004	1.4137 ± 0.0003
SP1-PDMS	1.4134 ± 0.0002	1.4137 ± 0.0002	1.4137 ± 0.0001	1.4143 ± 0.0002
SP2- PDMS	1.4135 ± 0.0001	1.4137 ± 0.0002	1.4137 ± 0.0001	1.4139 ± 0.0002

All the variations in RI in the non-irradiated state and after UV-A exposure are within the standard deviation and the stated accuracy (± 0.0003) of the refractometer. Despite a clear color change in the doped samples (Fig. 4a and b insets), the dopant concentration appears to have no effect on the RI values measured by this method. Only SP1-PDMS presented a measurable, albeit small, RI increase at 633 nm after UV irradiation. These results are reasonable considering that PDMS accounts for at least 99.95 wt% of the materials’ composition and that significant increases in refractive index have only been reported for SP1-doped PMMA at very high dopant concentrations, 20 to 50 wt%, in Refs. [55–58]. The increase in RI is associated with the formation of the dipolar MC species, consequently raising the dipolar density (and therefore the polarizability) of the material. In summary, both spiropyrans were responsive to UV light, preserving their photochromic properties when incorporated in PDMS and prepared as free-standing films. SP1-PDMS exhibited negligible negative photochromism and was less prone to photofatigue over the irradiation cycles.

### Characterization of the waveguides

**Fig. 5:**
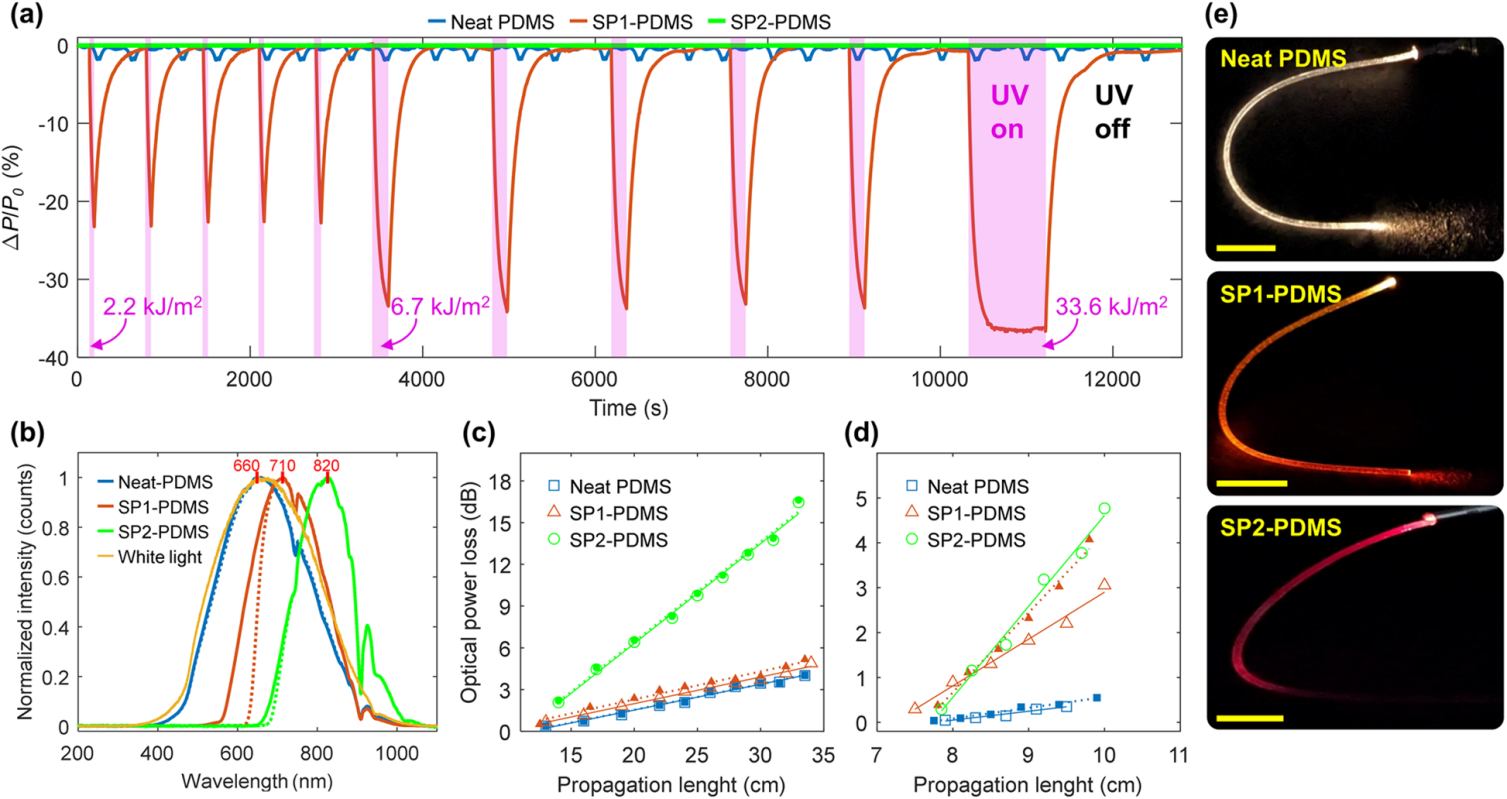
**a** Dynamic optical response of the waveguides to UV-A light under various exposure times. The pink shaded areas indicate the periods when the UV lamp was turned on. Non-shaded areas represent the recovery period in the dark (UV off). The first 5 cycles consisted of 1 min of UV exposure, followed by 5 cycles of 3 min and one cycle of 15 min. **b** Normalized optical transmission spectra of the white light source, neat PDMS, SP1- and SP2-PDMS before and immediately after UV exposure (solid and dotted lines, respectively). Representative data points and linear regression used to estimate the propagation loss **c** in white light and **d** at 633 nm, both before (solid lines and closed markers) and after (dotted lines and open markers) UV irradiation. **e** Images of the waveguides coupled to a white light source in the dark. Scale bars: 1 cm

The UV response of optical waveguides coupled to a He-Ne laser (633 nm) was investigated for different time periods, and consequently to different UV doses, as shown in Fig. 5a.

The percentage change in the transmitted optical power [∆*P*/*P*_0_ (%)] of the SP1-PDMS waveguide sharply decreased as soon as the UV light was turned on, indicating a fast response to UV exposure. It was followed by the optical power recovery immediately after it was turned off (Fig. 5a). The sharp reduction in optical transmission is ascribed to the formation of absorbing MC species, with a decay time constant of 42 ± 3 s. The latter is related to the thermally driven ring-closing reaction that yields the colorless SP form, with an average recovery time constant of 107 ± 6 s. Such decay time constant allows monitoring the cumulative UV dose with time and can be adjusted by varying the waveguide length as further discussed. The response was fully reversible under the tested conditions, regardless of the UV dose delivered to the SP1-PDMS waveguides. Meanwhile, the neat PDMS waveguides (tested for reference) presented an average variation of approximately 2% in the transmitted optical power because of the laser signal fluctuation and showed no measurable response to the UV radiation. The opacity of the SP2-PDMS waveguides for wavelengths up to 650 nm (Fig. 5b), owed to the negative photochromism, rendered a transmitted optical power near zero during the whole test. The high optical losses within the SP2-PDMS samples are also confirmed by the steep propagation losses shown in Fig. 5c and d, and the absence of optical power towards the end of the photographed waveguide (Fig. 5e).

The drop in SP1-PDMS transmitted optical power corroborates the narrowing of the transmission band observed after UV exposure, (orange curves in Fig. 5b), for which the power intensity at 633 nm was reduced by 84 ± 6% under UV irradiation. Meanwhile, neat PDMS and SP2-PDMS intensities decreased by only 0.3 ± 0.4% and 2 ± 1%, respectively. The output spectrum of the neat PDMS waveguide resembles that of the white light source whereas the SP-doped waveguides present transmission peaks shifted to the near-infrared range. All samples exhibited a reduction in optical intensity around 745 and 908 nm that can be ascribed to the stretching vibrations of the methyl groups (‒CH_3_) [59]. The described changes in the transmission spectra are in agreement with the reddish appearance of the SP-doped waveguides observed in Fig. 5e compared to the neat PDMS. The calculated propagation losses (in dB/cm) and bend losses in dB per unit of curvature (cm^−1^) for the waveguides are summarized in Table 3.

**Table 3:** Propagation and bend losses of neat and SP-doped PDMS waveguides, before and after UV irradiation

Sample	Propagation loss in white light (dB/cm)		Propagation loss at 633 nm (dB/cm)		Bend loss at 633 nm (dB∙cm)	
	Before UV irradiation	After UV irradiation	Before UV irradiation	After UV irradiation	Before UV irradiation	After UV irradiation
Neat PDMS	0.17 ± 0.02	0.18 ± 0.03	0.23 ± 0.03	0.23 ± 0.05	1.06 ± 0.06	Not measured
SP1-PDMS	0.19 ± 0.02	0.20 ± 0.01	1.04 ± 0.05	1.79 ± 0.05	1.51 ± 0.07	1.55 ± 0.10
SP2-PDMS	0.72 ± 0.01	0.73 ± 0.01	2.05 ± 0.10	Not measurable	Not measurable	Not measurable

We note that the measured neat PDMS propagation losses are in agreement with similar demonstrations reported in the literature [60, 61]. Doping PDMS with the spiropyrans SP1 or SP2 increased the waveguides’ propagation losses, particularly at 633 nm in the non-irradiated state. Absorption and scattering losses are expected in the presence of finely dispersed dyes that act as absorbing and scattering centers. The SP2-PDMS waveguides presented large losses at 633 nm, and measurements in the UV-irradiated state were impractical because of the poor signal-to-noise ratio. Besides, a bent optical waveguide will present radiation and transition losses due to changes in curvature that ultimately results in increased propagation loss [61, 62]. The neat PDMS waveguides presented an average bend loss of 1.06 dB∙cm at 633 nm as shown in Table 3 (more details in Fig. S5). When doped with SP1, the optical losses upon bending increased by approximately 0.45 dB∙cm (Table 3).

**Fig. 6:**
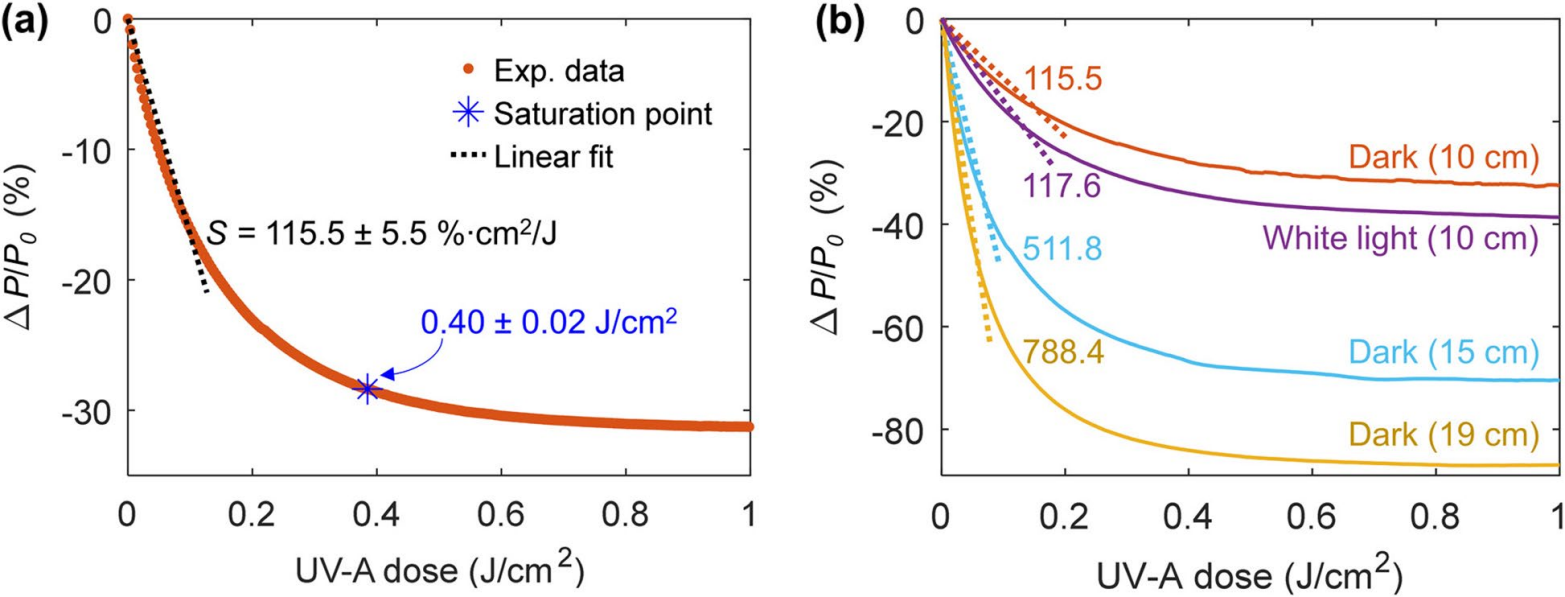
**a** Representative SP1-PDMS experimental data and best-fit line used to estimate the sensitivity in the linear range of the SP1-PDMS curve response. The blue asterisk indicates the saturation point. **b** Experimental data and best-fit lines for SP1-PDMS at different lengths, tested in the dark and in white light. Numbers indicate the average sensitivity in %·cm^2^/J calculated from at least three independent measurements

The relationship between the delivered UV dose in J/cm^2^ and the SP1-PDMS percentage change in the laser’s transmitted optical power [∆*P*/*P*_0_ (%)] has an exponential form. Because of the nonlinear response, the sensor’s sensitivity (*S*), i.e., the percentage change in transmitted optical power per unit change in UV-A radiation dose, was estimated by the best-fit slope in the linear range at 115.5 ± 5.5%∙cm^2^/J (Fig. 6a). The saturation point is defined as the UV dose at which no more significant changes in transmitted optical power occur. It was calculated at 0.40 ± 0.02 J/cm^2^ for 10-cm long waveguides tested in the dark, which corresponds to a percentage power change of 29.5 ± 3%. The UV-A dose range detected by the sensor is within the magnitudes used in UV plant supplementation [16], photopolymerization [12], phototherapy [10, 11], and other UV colorimetric dosimeters [27, 30, 37, 38]. The detection limit, typically considered as three times the noise signal, was found to be 3.2 ± 1.1%, which corresponds to 0.03 ± 0.01 J/cm^2^. Moreover, these sensor parameters at various bend radii were consistent and independent of bending (Table S2 in SI), as similarly reported by Kee et al. [61]. This means that the SP-doped PDMS waveguides maintain their ability to be used for UV sensing even under significant bending.

Increasing the optical path length is going to affect the sensor performance as the rate of change in transmitted optical power is proportional to the distance traveled by light, as given by Beer-Lambert’s law. Indeed, a sharp increase in sensitivity was observed for longer waveguides as shown in Fig. 6b as more molecules could interact with light along the optical path. For the tested waveguide lengths, all the other calculated parameters varied linearly (see details in Table S2, Fig. S6). By increasing the waveguide length, ∆*P*/*P*_0_ (%) reaches larger negative values. Based on a linear fit of the experimental data given in Fig. S6d, we estimate a maximum SP1-PDMS waveguide length of 21 cm, when it would reach a power change near −100%. The competing effect of the white light on the SP to MC conversion was observed through changes in some of the sensor parameters. Although the average sensitivity (Fig. 6b) and the decay time varied within the standard deviations under white light illumination, the recovery time was 15 s faster on average (Table S2). We note that under the tested conditions, the white light did not hinder the response of the SP1-PDMS waveguides and could even be used to speed up the sensor’s recovery time.

**Fig. 7:**
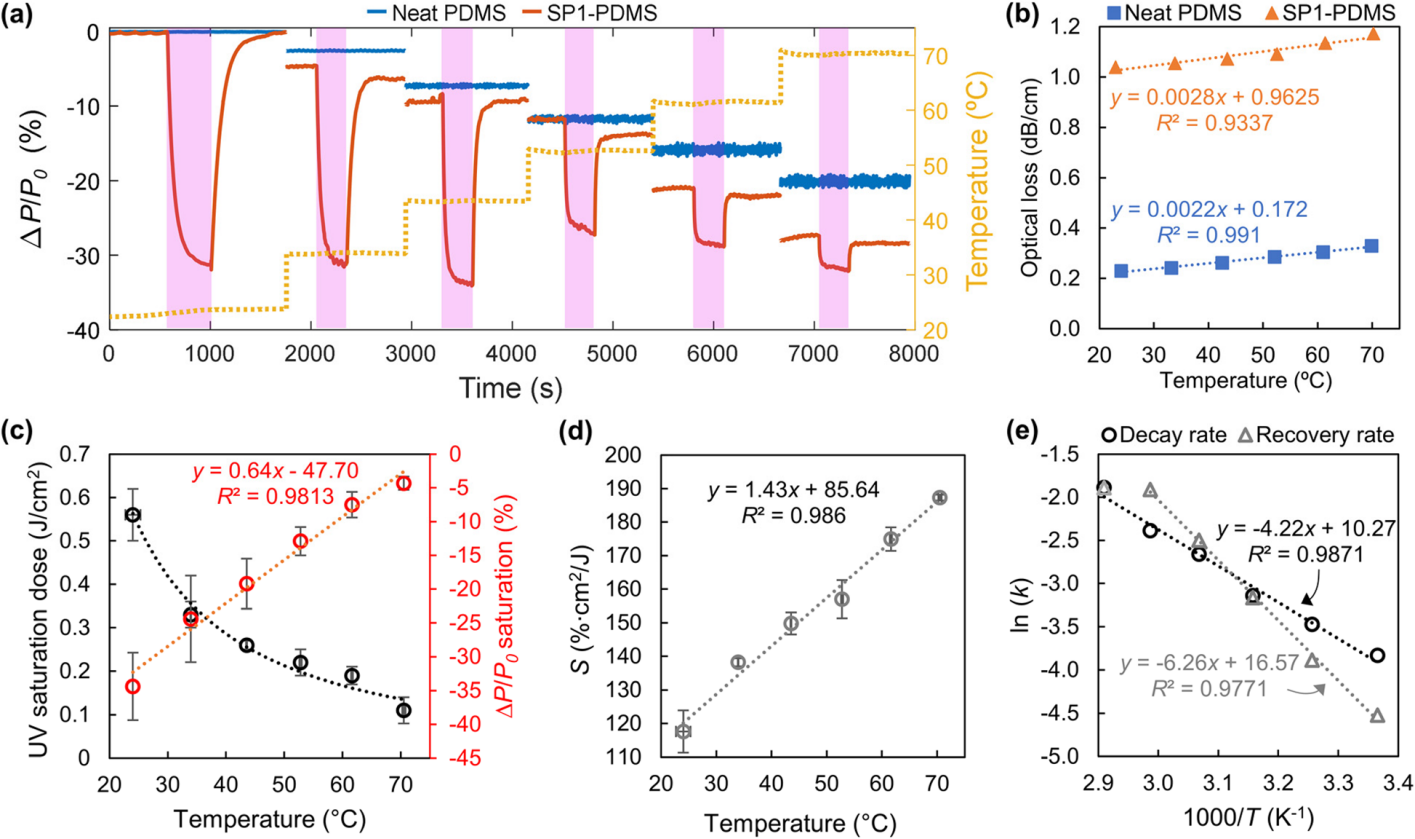
**a** Representative SP1-PDMS percentage response change with temperature and time. **b** Representative plot of optical loss, **c** UV saturation dose and percentage power at saturation, and **d** sensitivity, as a function of temperature. **e** Arrhenius plot with the decay and recovery rate constants (see Table S2 for details). Error bars indicate the sample standard deviation in separate measurements performed on three waveguides

Temperature-induced optical loss has been observed in polymer optical waveguides attributed to changes in refractive index (RI) and waveguide dimensions [63, 64]. This behavior is expected given that polymers have a negative linear correlation between thermo-optic and thermal expansion coefficients [65]. Figure 7a depicts a representative plot of the percentage change in transmitted optical power over time at different testing temperatures for the waveguides under test. Figure 7b–d show how several key parameters evolve as a function of temperature.

The temperature-induced optical loss of the neat and SP1-PDMS waveguides was 0.0022 ± 0.0001 and 0.0027 ± 0.0001 (dB/cm)/℃, respectively (Fig. 7b), and was shown to be reversible. PDMS is known to exhibit very large negative thermo-optic [59, 66], and positive thermal expansion coefficients [67–70], which can account for the former behavior. More specifically, the change in material density that occurs as a result of the waveguide’s dimensional expansion is accompanied by a RI drop. Furthermore, the expansion causes additional mechanical strain owing to mismatches in thermo-mechanical properties of the materials used for the connections, all of which contribute to the observed optical losses.

When doped with SP1, temperature will additionally affect the photoswitching reaction rates as the likelihood of molecular vibration and bond cleavage increases. Assuming that the rates of optical power change are related to the number of MC species coexisting in equilibrium with SP ones, the rise in temperature will shift the reversible reaction equilibrium to new values. Consequently, the sensor parameters will change, as shown in Fig. 7c and d. Furthermore, the decay and recovery rate constants increased with temperature (Fig. 7e, Table S2) following the Arrhenius type behavior, and in agreement with previous studies on spiropyrans in liquid and solid matrices [71, 72]. The turning point between 40 and 50 ℃ marks the approximate temperature at which the recovery rate becomes faster than the response rate. At 70 ℃ both rates are very similar, 0.15 s^−1^, and the response to UV light at higher temperatures is greatly reduced, setting the limit of the sensor response.

## Conclusion

In this work, physical doping was exploited as a straightforward approach for preparing PDMS doped with spiropyrans (SP) as a novel material platform for the reversible UV-induced optical changes in flexible optical waveguides. Using very small levels (0.05 wt%) of SP doping, the fabricated free-standing films and waveguides exhibited reversible UV response and good resistance to photofatigue, while incurring negligible optical losses (without UV excitation, white light source) compared to their neat PDMS counterparts. We further showed that adequate selection of the spiropyran derivative is critical for minimizing optical losses caused by negative photochromism in a polar matrix such as PDMS, along with the appropriate selection of the optical source (i.e., laser) used for optical sensing. The prepared SP-PDMS waveguides presented a UV response dependent on temperature, waveguide length, and white light, which can be further engineered to tailor the UV sensing performance in specific application scenarios. These results are relevant to elastomeric optics, smart optical materials, optical waveguide sensors, and UV sensing.

### Supplementary Information

Supplementary Material 1.

## Data Availability

The data supporting the findings of this study are available from the corresponding author upon reasonable request.

## References

[1] IARC Working Group on the Evaluation of Carcinogenic Risks to Humans: IARC Monographs on the Evaluation of Carcinogenic Risks to Humans. Volume 100. A Review of Human Carcinogens. Part D: Radiation. International Agency for Research on Cancer, Lyon (2012)

[2] Cherrie JW, Cherrie MPC (2022). Workplace exposure to UV radiation and strategies to minimize cancer risk. Br. Med. Bull..

[3] McKenzie RL, Aucamp PJ, Bais AF, Björn LO, Ilyas M, Madronich S (2011). Ozone depletion and climate change: impacts on UV radiation. Photochem. Photobiol. Sci..

[4] Barnes PW, Robson TM, Neale PJ, Williamson CE, Zepp RG, Madronich S, Wilson SR, Andrady AL, Heikkilä AM, Bernhard GH, Bais AF, Neale RE, Bornman JF, Jansen MAK, Klekociuk AR, Martinez-Abaigar J, Robinson SA, Wang QW, Banaszak AT, Häder DP, Hylander S, Rose KC, Wängberg SÅ, Foereid B, Hou WC, Ossola R, Paul ND, Ukpebor JE, Andersen MPS, Longstreth J, Schikowski T, Solomon KR, Sulzberger B, Bruckman LS, Pandey KK, White CC, Zhu L, Zhu M, Aucamp PJ, Liley JB, McKenzie RL, Berwick M, Byrne SN, Hollestein LM, Lucas RM, Olsen CM, Rhodes LE, Yazar S, Young AR (2022). Environmental effects of stratospheric ozone depletion, UV radiation, and interactions with climate change: UNEP Environmental Effects Assessment Panel, Update 2021. Photochem. Photobiol. Sci..

[5] Fernández-Marchante CM, Souza FL, Millán M, Lobato J, Rodrigo MA (2021). Does intensification with UV light and US improve the sustainability of electrolytic waste treatment processes?. J. Environ. Manage..

[6] EPA 832-F-99-064 Wastewater Technology Fact Sheet Ultraviolet Disinfection. United States Environmental Protection Agency (1999)

[7] Raeiszadeh M, Adeli B (2020). A critical review on ultraviolet disinfection systems against COVID-19 outbreak: applicability, validation, and safety considerations. ACS Photonics.

[8] Ramos CCR, Roque JLA, Sarmiento DB, Suarez LEG, Sunio JTP, Tabungar KIB, Tengco GSC, Rio PC, Hilario AL (2020). Use of ultraviolet-C in environmental sterilization in hospitals: a systematic review on efficacy and safety. Int. J. Health Sci. (Qassim).

[9] Bagheri A, Jin J (2019). Photopolymerization in 3D printing. ACS Appl. Polym. Mater..

[10] Weller RB, Macintyre IM, Melville V, Farrugia M, Feelisch M, Webb DJ (2022). The effect of daily UVA phototherapy for 2 weeks on clinic and 24-h blood pressure in individuals with mild hypertension. J. Hum. Hypertens..

[11] Sidbury R, Davis DM, Cohen DE, Cordoro KM, Berger TG, Bergman JN, Chamlin SL, Cooper KD, Feldman SR, Hanifin JM, Krol A, Margolis DJ, Paller AS, Schwarzenberger K, Silverman RA, Simpson EL, Tom WL, Williams HC, Elmets CA, Block J, Harrod CG, Begolka WS, Eichenfield LF (2014). Guidelines of care for the management of atopic dermatitis. J. Am. Acad. Dermatol..

[12] Childress KK, Kim K, Glugla DJ, Musgrave CB, Bowman CN, Stansbury JW (2019). Independent control of singlet oxygen and radical generation via irradiation of a two-color photosensitive molecule. Macromolecules.

[13] van der Laan HL, Burns MA, Scott TF (2019). Volumetric photopolymerization confinement through dual-wavelength photoinitiation and photoinhibition. ACS Macro Lett..

[14] Schlotthauer T, Nitsche J, Middendorf P (2021). Evaluation of UV post-curing depth for homogenous cross-linking of stereolithography parts. Rapid Prototyping J..

[15] Zhang Y, Sun X, Aphalo PJ, Zhang Y, Cheng R, Li T (2024). Ultraviolet-A1 radiation induced a more favorable light-intercepting leaf-area display than blue light and promoted plant growth. Plant Cell Environ..

[16] Mariz-Ponte N, Mendes RJ, Sario S, Correia CV, Correia CM, Moutinho-Pereira J, Melo P, Dias MC, Santos C (2021). Physiological, biochemical and molecular assessment of UV-A and UV-B supplementation in solanum lycopersicum. Plants.

[17] Rabek JF (1995). Polymer photodegradation.

[18] Rajan A, Kaur G, Paliwal A, Yadav HK, Gupta V, Tomar M (2014). Plasmonic assisted enhanced photoresponse of metal nanoparticle loaded ZnO thin film ultraviolet photodetectors. J. Phys. D Appl. Phys..

[19] Sang L, Liao M, Sumiya M (2013). A comprehensive review of semiconductor ultraviolet photodetectors: from thin film to one-dimensional nanostructures. Sensors (Basel).

[20] Ye Q, Zhang X, Yao R, Luo D, Liu X, Zou W, Guo C, Xu Z, Ning H, Peng J (2021). Research and progress of transparent, flexible tin oxide ultraviolet photodetector. Crystals (Basel).

[21] Zhou X, Lu Z, Zhang L, Ke Q (2023). Wide-bandgap all-inorganic lead-free perovskites for ultraviolet photodetectors. Nano Energy.

[22] Zou W, Sastry M, Gooding JJ, Ramanathan R, Bansal V (2020). Recent advances and a roadmap to wearable UV sensor technologies. Adv. Mater. Technol..

[23] Kanellis VG (2019). Ultraviolet radiation sensors: a review. Biophys. Rev..

[24] Huang X, Chalmers AN (2021). Review of wearable and portable sensors for monitoring personal solar UV exposure. Ann. Biomed. Eng..

[25] Zhang Z, Geng Y, Cao S, Chen Z, Gao H, Zhu X, Zhang X, Wu Y (2022). Ultraviolet photodetectors based on polymer microwire arrays toward wearable medical devices. ACS Appl. Mater. Interfaces.

[26] Henning A, Downs JN, Vanos JK (2022). Wearable ultraviolet radiation sensors for research and personal use. Int. J. Biometeorol..

[27] Zhang P, Carrillo Segura S, Boldini A, Di Trolio P, Ohanian OJ, Porfiri M (2021). A photochromic nylon webbing for ultra-violet light sensing. Smart Mater. Struct..

[28] Wang W, Tian S, Lu J, Zheng Y, Yan Z, Wang D (2021). Highly sensitive photoresponsive polyamide 6 nanofibrous membrane containing embedded spiropyran. J. Mater. Sci..

[29] Bao B, Fan J, Wang W, Yu D (2020). Photochromic cotton fabric prepared by spiropyran-ternimated water polyurethane coating. Fibers Polym..

[30] Araki H, Kim J, Zhang S, Banks A, Crawford KE, Sheng X, Gutruf P, Shi Y, Pielak RM, Rogers JA (2017). Materials and device designs for an epidermal UV colorimetric dosimeter with near field communication capabilities. Adv. Funct. Mater..

[31] Qi, Y., Zheng, J.: An Azo-PDMS-based wearable UV sensor with the optimized photo response mode for dual sensing and synchronous detection. Sci. China Technol. Sci. **65**, 179–190 (2021)

[32] Chen Y, Cao Z, Zhang J, Liu Y, Yu D, Guo X (2022). Wearable ultraviolet sensor based on convolutional neural network image processing method. Sens. Actuators A Phys..

[33] Fan S, Lam Y, Yang J, Bian X, Xin JH (2022). Development of photochromic poly(azobenzene)/PVDF fibers by wet spinning for intelligent textile engineering. Surf. Interfaces.

[34] Fang W, Sairanen E, Vuori S, Rissanen M, Norrbo I, Lastusaari M, Sixta H (2021). UV-sensing cellulose fibers manufactured by direct incorporation of photochromic minerals. ACS Sustain. Chem. & Eng..

[35] Finny AS, Jiang C, Andreescu S (2020). 3D printed hydrogel-based sensors for quantifying UV exposure. ACS Appl. Mater. Interfaces.

[36] Lee ME, Armani AM (2016). Flexible UV exposure sensor based on UV responsive polymer. ACS Sens..

[37] Yang Z, Zhao J, Liang C, Jiang H (2023). Materials and device design for epidermal UV sensors with real-time, skin-color specific, and naked-eye quasi-quantitative monitoring capabilities. Adv. Mater. Technol..

[38] Yimyai T, Crespy D, Pena-Francesch A (2023). Self-healing photochromic elastomer composites for wearable UV-sensors. Adv. Funct. Mater..

[39] Chen GY, Wang Z (2015). Towards extremely sensitive ultraviolet-light sensors employing photochromic optical microfiber. J. Sens..

[40] Ock K, Jo N, Kim J, Kim S, Koh K (2001). Thin film optical waveguide type UV sensor using a photochromic molecular device, spirooxazine. Synth. Met..

[41] Song, I.S., Kim, C.Y., Han, A.R., Yoo, J.S., Lee, S.Y., Kim, H.K., Ahn, T.J.: Azobenzene polymer waveguide for UV sensors. In: 2012 Photonics Global Conference (PGC). pp. 1–3. IEEE, Singapore (2012)

[42] Yoon JK, Seo GW, Cho KM, Kim ES, Kim SH, Kang SW (2003). Controllable in-line UV sensor using a side-polished fiber coupler with photofunctional polymer. IEEE Photonics Technol. Lett..

[43] Kortekaas L, Browne WR (2019). The evolution of spiropyran: fundamentals and progress of an extraordinarily versatile photochrome. Chem. Soc. Rev..

[44] Klajn R (2014). Spiropyran-based dynamic materials. Chem. Soc. Rev..

[45] Crano, J.C., Guglielmetti, R.J.: eds.: Chapter 2: photodegradation of organic photochromes. In: Organic Photochromic and Thermochromic Compounds Volume 2: Physicochemical Studies, Biological Applications, and Thermochromism, pp. 65–166. Kluwer Academic Publishers, New York (2002)

[46] Virlogeux F, Bianchini D, Delor-Jestin F, Baba M, Lacoste J (2004). Evaluation of cross-linking after accelerated photo-ageing of silicone rubber. Polym. Int..

[47] Stevenson I, David L, Gauthier C, Arambourg L, Davenas J, Vigier G (2001). Influence of SiO_2_ fillers on the irradiation ageing of silicone rubbers. Polymer (Guildf.).

[48] Minkin VI (2004). Photo-, thermo-, solvato-, and electrochromic spiroheterocyclic compounds. Chem. Rev..

[49] Berman E, Fox RE, Thomson FD (1959). Photochromic spiropyrans. I. The effect of substituents on the rate of ring closure. J. Am. Chem. Soc..

[50] The Dow Chemical Company: Technical Data Sheet: SYLGARD^TM^ 184 Silicone Elastomer (2017)

[51] Yu CU, Mark JE (1974). Specific solvent effects in swollen polymer networks. Macromolecules.

[52] Kim D, Kim SH, Park JY (2019). Floating-on-water fabrication method for thin polydimethylsiloxane membranes. Polymers (Basel).

[53] Nam YS, Yoo I, Yarimaga O, Park IS, Park DH, Song S, Kim JM, Lee CW (2014). Photochromic spiropyran-embedded PDMS for highly sensitive and tunable optochemical gas sensing. Chem. Commun. (Camb.).

[54] Tian W, Tian J (2014). An insight into the solvent effect on photo-, solvato-chromism of spiropyran through the perspective of intermolecular interactions. Dyes Pigments.

[55] Qiao C, Zhang C, Zhou Z, Dong H, Du Y, Yao J, Zhao YS (2020). A photoisomerization-activated intramolecular charge-transfer process for broadband-tunable single-mode microlasers. Angew. Chem. Int. Ed..

[56] Wallikewitz BH, Nikiforov GO, Sirringhaus H, Friend RH (2012). A nanoimprinted, optically tuneable organic laser. Appl. Phys. Lett..

[57] Lin L, Wang M, Wei X, Peng X, Xie C, Zheng Y (2016). Photoswitchable Rabi splitting in hybrid plasmon–waveguide modes. Nano Lett..

[58] Zheng YB, Kiraly B, Cheunkar S, Huang TJ, Weiss PS (2011). Incident-angle-modulated molecular plasmonic switches: a case of weak exciton–plasmon coupling. Nano Lett..

[59] Cai, D., Heise, H.M.: Spectroscopic aspects of polydimethylsiloxane (PDMS) used for optical waveguides. In: Koleżyński, A., Król, M. (eds.) Molecular Spectroscopy—Experiment and Theory. Challenges and Advances in Computational Chemistry and Physics. pp. 401–425. Springer, Switzerland (2019)

[60] Sharma K, Morlec E, Valet S, Camenzind M, Weisse B, Rossi RM, Sorin F, Boesel LF (2023). Polydimethylsiloxane based soft polymer optical fibers: from the processing-property relationship to pressure sensing applications. Mater. Des..

[61] Kee JS, Poenar DP, Neuzil P, Yobas L (2008). Monolithic integration of poly(dimethylsiloxane) waveguides and microfluidics for on-chip absorbance measurements. Sens. Actuators B Chem..

[62] Papakonstantinou I, Wang K, Selviah DR, Fernández FA (2007). Transition, radiation and propagation loss in polymer multimode waveguide bends. Opt. Express.

[63] Suar M, Baran M, Günther A, Roth B (2020). Combined thermomechanical and optical simulations of planar-optical polymer waveguides. J. Opt..

[64] Günther A, Baran M, Garg R, Roth B, Kowalsky W (2022). Analysis of the thermal behavior of self-written waveguides. Opt. Lasers Eng..

[65] Zhang Z, Zhao P, Lin P, Sun F (2006). Thermo-optic coefficients of polymers for optical waveguide applications. Polymer (Guildf.).

[66] Zhu Z, Liu L, Liu Z, Zhang Y, Zhang Y (2017). Surface-plasmon-resonance-based optical-fiber temperature sensor with high sensitivity and high figure of merit. Opt. Lett..

[67] Information about Dow Corning brand Silicone Encapsulants. Dow Corning Corporation, USA (2005)

[68] Gupta NS, Lee KS, Labouriau A (2021). Tuning thermal and mechanical properties of polydimethylsiloxane with carbon fibers. Polymers (Basel).

[69] Müller A, Wapler MC, Wallrabe U (2019). A quick and accurate method to determine the Poisson’s ratio and the coefficient of thermal expansion of PDMS. Soft Matter.

[70] Zhang G, Sun Y, Qian B, Gao H, Zuo D (2020). Experimental study on mechanical performance of polydimethylsiloxane (PDMS) at various temperatures. Polym. Test..

[71] Lin JS (2003). Interaction between dispersed photochromic compound and polymer matrix. Eur. Polym. J..

[72] Sworakowski J, Janus K, Nešpůrek S (2005). Kinetics of photochromic reactions in condensed phases. Adv. Colloid Interface Sci..

