# Supplementary material for: Novel elastomeric spiropyran-doped poly(dimethylsiloxane) optical waveguide for UV sensing
Source: Front Optoelectron. 2024 Jul 15;17(1):21. doi: 10.1007/s12200-024-00124-4 (PMC11250767; doi:10.1007/s12200-024-00124-4)
Supplement: Supplementary file 1 — Supplementary Material 1. [file 12200_2024_124_MOESM1_ESM.docx]

**Supplementary Information (SI)**

Novel elastomeric spiropyran-doped poly(dimethylsiloxane) optical waveguide for UV sensing

**Camila Aparecida Zimmermann**^1^**, Koffi Novignon Amouzou**^1^**, Dipankar Sengupta**^1^**, Aashutosh Kumar**^1^**, Nicole Raymonde Demarquette**^2^**, Bora Ung**^1*^

^1^ Department of Electrical Engineering, École de Technologie Supérieure, Montreal, QC, H3C 1K3, Canada

^2^ Department of Mechanical Engineering, École de Technologie Supérieure, Montreal, QC, H3C 1K3, Canada

‎^*^Corresponding author: [bora.ung@etsmtl.ca](mailto:bora.ung@etsmtl.ca)

# Spectra of the light sources

The normalized spectra of the light sources used in this work are given in Fig. S1. They were collected at different spots along the setup to check for consistency. The white light source HR-2000-HP was directly connected to the spectrometer with the QP600-1-SR fiber.

| **** |
| --- |

**Fig. S1** Normalized intensity spectra of the **a** white light source HL-2000-HP Ocean Optics, main peak at 660 nm, **b** UV-A light source F15T8/BL Hitachi, main peak at 370 nm, and **c** LED white lamp PLYB1002D, 2700K Luminus Inc., main peak at 603 nm

# Optical microscopy of free-standing films

The micrographs presented in Fig. S2 are representative of the five films evaluated per composition with a polarized optical microscope (POM) Olympus BX51.

| 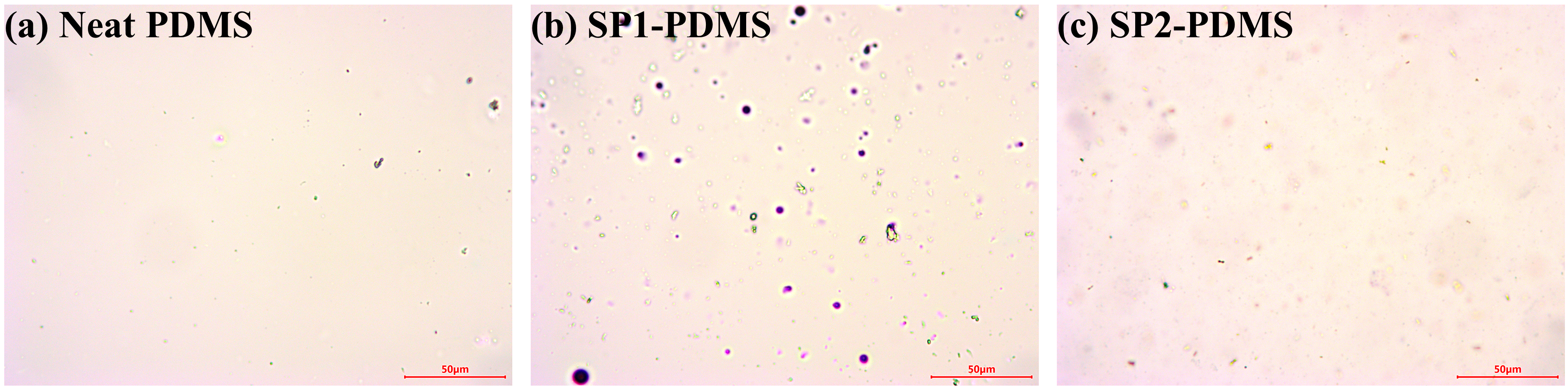 |
| --- |

**Fig. S2** Micrographs of the free-standing films under a POM. Scale bars: 50 µm

SP1 is characterized by round purple particles with d_50_ of 1.4 ± 0.1 µm, as depicted in Fig. S2b. Meanwhile, SP2 is found to be very fine particles with random shapes and d_50_ of 0.9 ± 0.1 µm (Fig. S2c). In contrast, only some dust particles are observed in the neat PDMS films.

# Complementary discussion on energy levels

The polarity of the surrounding medium has been shown to have an effect on the spiropyran (SP) electronic spectrum [1, 2]. PDMS is a polar polymer with a dipole moment (*µ*) of 0.67 Debye [3]. It has a glass transition temperature (*T_g_*) of around −120 ºC and a melting temperature (*T_m_*) near −45 ºC [4]. Additionally, residual chloroform, calculated at 4 wt%, is a polar solvent that may interact with the spiropyrans via hydrogen bonding [5] and dipole-dipole interactions as well. Based on the transition temperatures and accounting for the residual solvent, it is assumed that enough free volume is provided for SP to merocyanine (MC) conversion in the solid state and that the MC form is stabilized by strong intermolecular interactions with the PDMS chains and chloroform molecules, as illustrated in Fig. S3a. The qualitative scheme given in Fig. S3b illustrates the differences in MC energy levels in a polar and non-polar medium. One should note that the SP-doped PDMS energy levels and their indirectly related color appearance are the average result of SP and MC species in different proportions and the complex interplay of intermolecular interactions between themselves and the matrix.

| 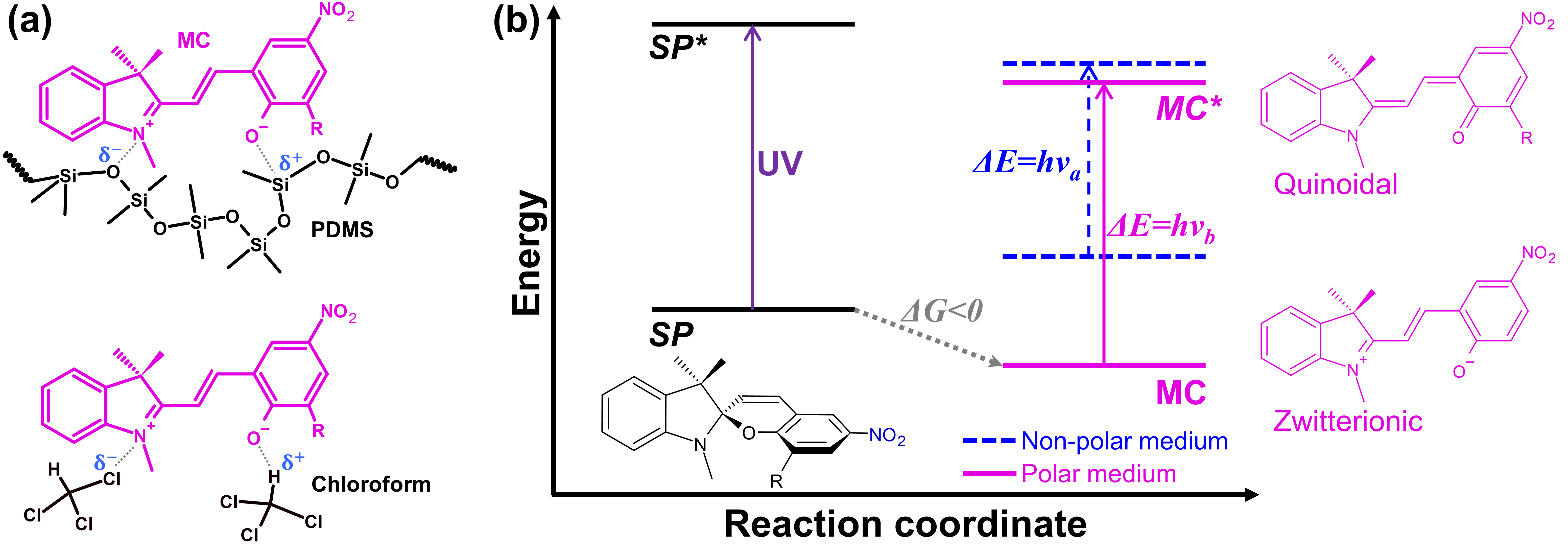 |
| --- |

**Fig. S3 a** Representation of the dipole-dipole interaction between an MC molecule and a PDMS chain (top), and dipole-dipole and hydrogen bonding with two chloroform molecules (bottom). **b** A qualitative energy diagram of the SP and MC states in polar and non-polar mediums

The interactions between MC and the polymer matrix will influence the energy levels of the MC ground and excited states differently. The MC excited state has a lower dipole moment due to the conjugation effect that redistributes the electrons of the π-system and is well represented by the MC quinoidal form [2, 6, 7]. A polar medium (PDMS and chloroform) stabilizes and decreases more sharply the energy level of the MC ground state (zwitterionic form), which is more polar and prone to dipole-dipole interactions. As a consequence, the energy level of the MC ground state is lowered below the SP level when dispersed in PDMS and chloroform. Hence, the SP to MC conversion is spontaneous in the dark, i.e., the Gibbs free energy (Δ*G*) is lower than 0 [1].

By decreasing the MC ground state, the energy bandgap (Δ*E*) between the MC ground and excited states increases. More energetic photons are expected to be absorbed upon UV irradiation, resulting in a blue shift in the *λ_MC_* compared to the spectra of the same SPs dissolved in a non-polar medium, i.e., ΔE_a_<ΔE_b_ (Fig. S3b) [1, 2, 8]. To test this hypothesis, we have doped two other polymers typically used in the fabrication of optical waveguides: a polar polymer, poly(methyl methacrylate) (PMMA), and a non-polar one, polystyrene (PS) (see Table S1).

Spiropyran-doped polymer films were prepared by spin-coating. Briefly, the polymer-doped solutions were prepared by mixing 1 wt% of spiropyran on a dry basis at different concentrations in toluene to produce films of controlled thickness. A volume of 500 μL of polymer-doped solutions was dispensed on glass substrates (Fischer Premium microscope glass slides, 25 × 75 × 1 mm) previously cleaned with ethanol and acetone. After static deposition, the substrate was spun for 60 s at 1500 rpm, followed by a final step at 100 rpm for 5 s. The spin-coating was conducted at room conditions (23 ± 1 ºC, 25 ± 10 % R.H.) in a spin-coater (Ossila, UK). The samples were left to dry in the dark until they reached a roughly constant weight (for at least a week) before test. The average film thickness of 1.6 ± 0.2 µm was estimated based on the film weight and the area covered by it. The films’ final residual solvent has been estimated at 1.5 ± 0.1 wt% based on TGA tests carried out in Argon from 50–125 °C at 10 K/min. The *T_g_* of the polymers, as received, was determined by DSC using a PerkinElmer Pyris 1 device. The results presented in Table S1 were duplicated and taken at the half heat capacity (half *Cp*) from the second cooling cycle at 5 K/min in N_2_.

**Table S1** The molecular structure, manufacturer, glass transition temperature, dipole moment and transmittance spectra evolution of the used materials

| **Molecular structure** | **Description** | ***T_g_* (ºC)** | ***µ* (D)**^a^ | ***λ_MC_* (nm)**^b^ | |
| --- | --- | --- | --- | --- | --- |
|  |  |  |  | **SP1** | **SP2** |
| 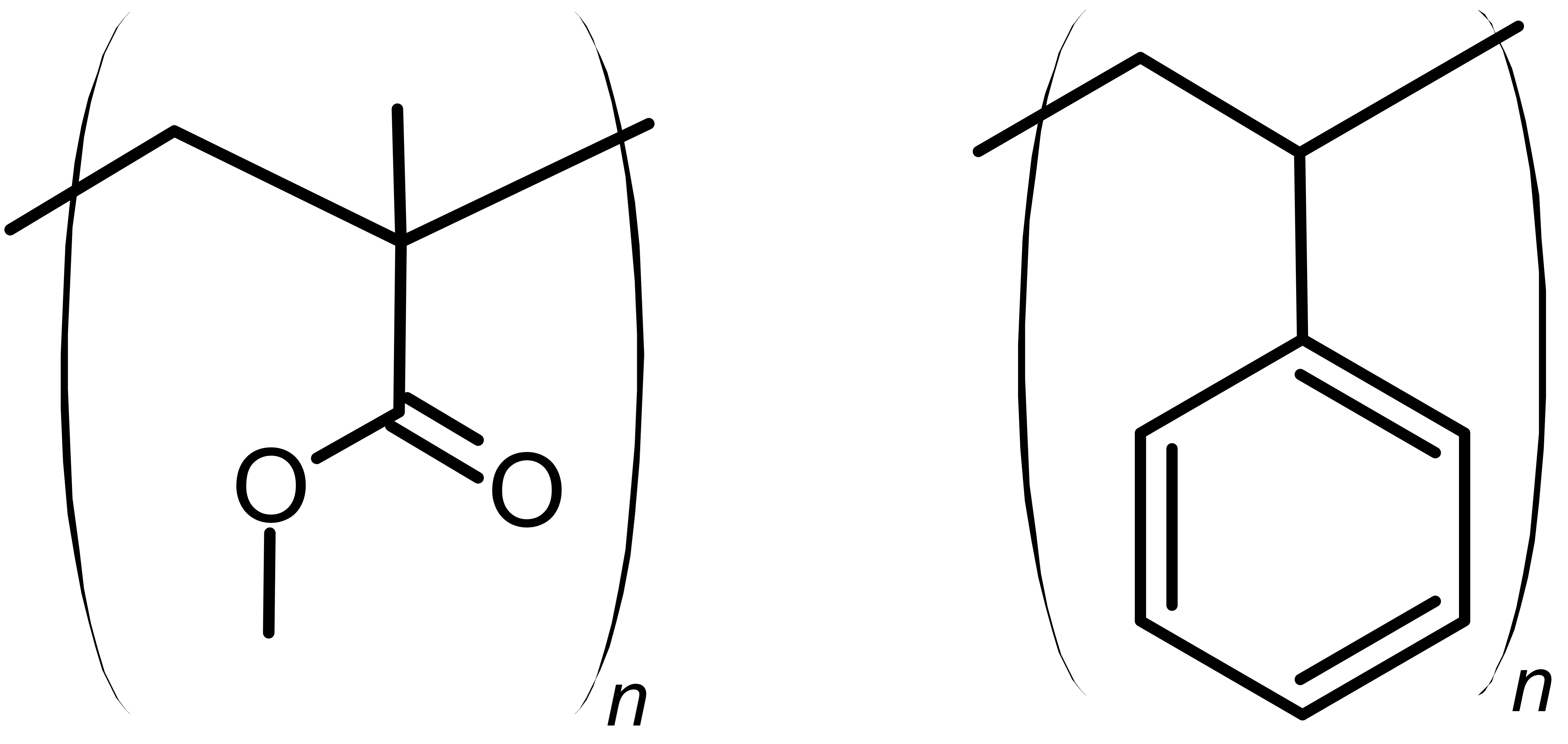 | Poly(methyl methacrylate) (PMMA), Evonik | 96 ± 0.2 | 0.7 | 585 | 600 |
| 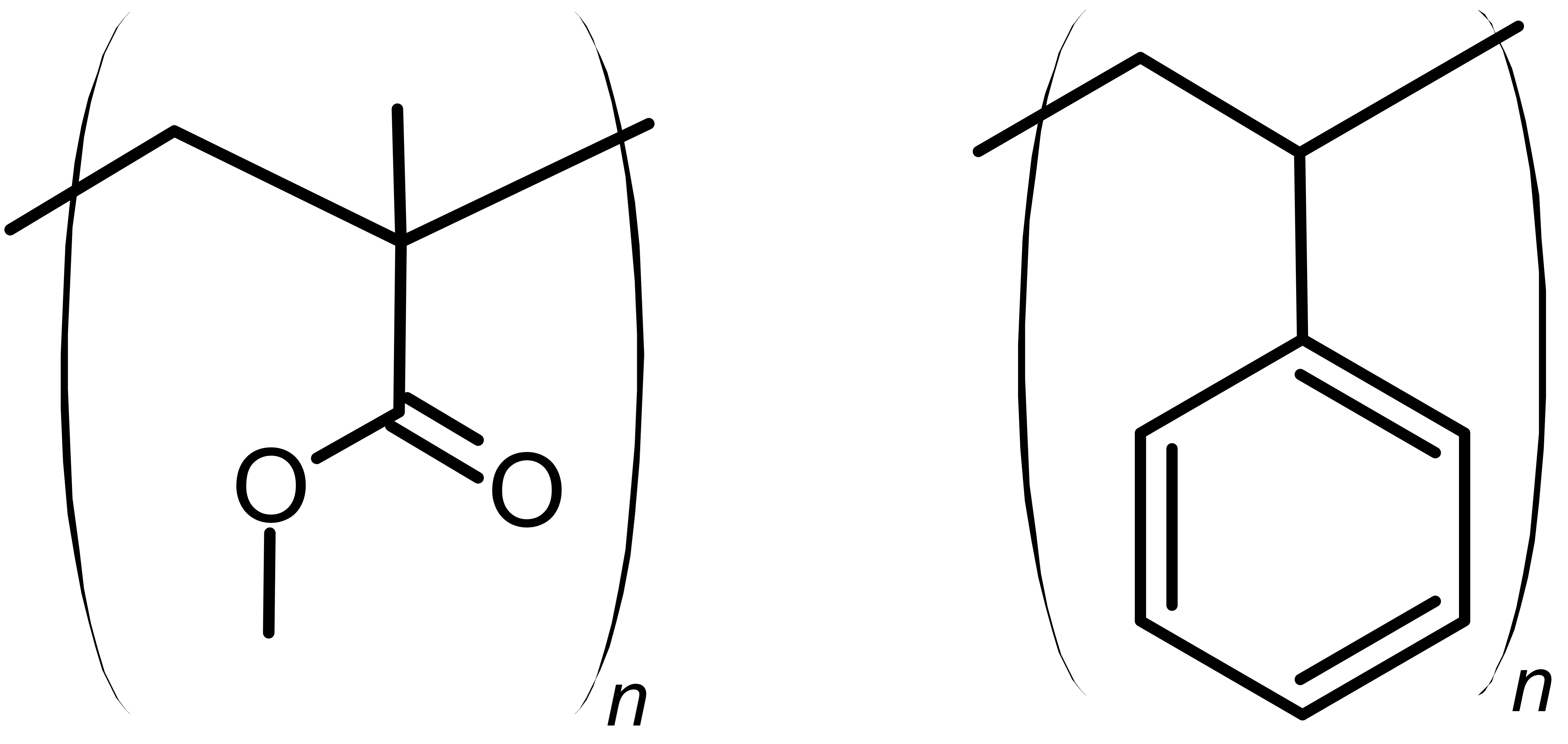 | Polystyrene Styron 685D (PS), AmSty | 102 ± 1 | 0.1 | 607 | 625 |
| 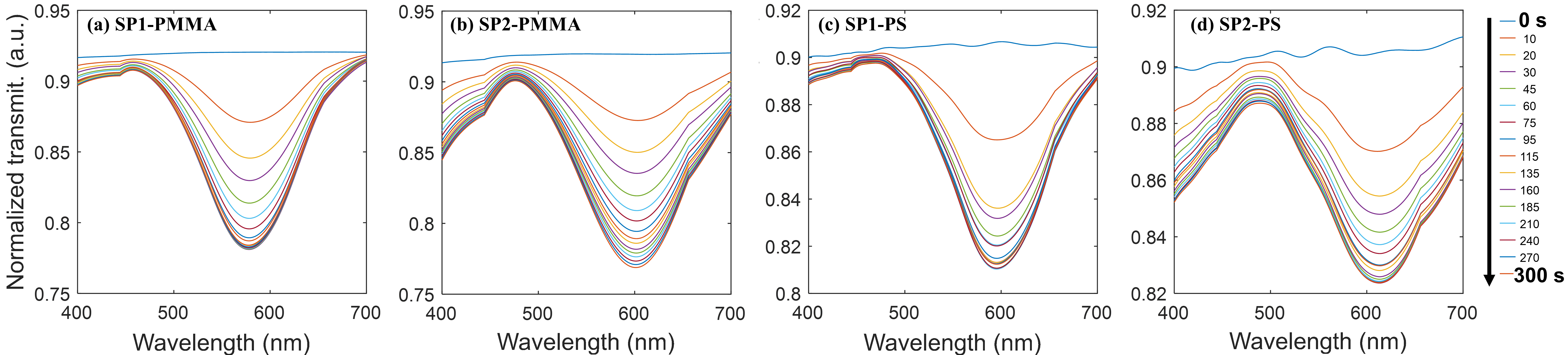 | | | | | |

^a^ Experimental dipole moment at 298 K taken from [3]. ^b^After UV-A irradiation for 5 min.

In fact, SP-doped PDMS and PMMA MC peaks are blue-shifted compared to SP-doped PS, which is in good agreement with Lin (2003) [8]. Moreover, no absorption peaks after dark storage for 24 h and before UV irradiation are observed on the doped PMMA and PS samples spectra (Table S1). Interestingly, the negative photochromism is not present in SP-PMMA samples, probably because of the low chain mobility for the spontaneous SP-to-MC conversion, marked by the high *T_g_* of PMMA.

# Transmittance evolution with irradiation cycles

The transmittance evolution of the SP1- and SP2-PDMS free-standing films evaluated over the course of 23 UV-dark cycles is shown in Fig. S4, with the least square linear fit that best described the experimental data. Those equations were used to estimate the number of cycles necessary to reach a 50% increase in transmittance due to photofatigue (see main text).

| 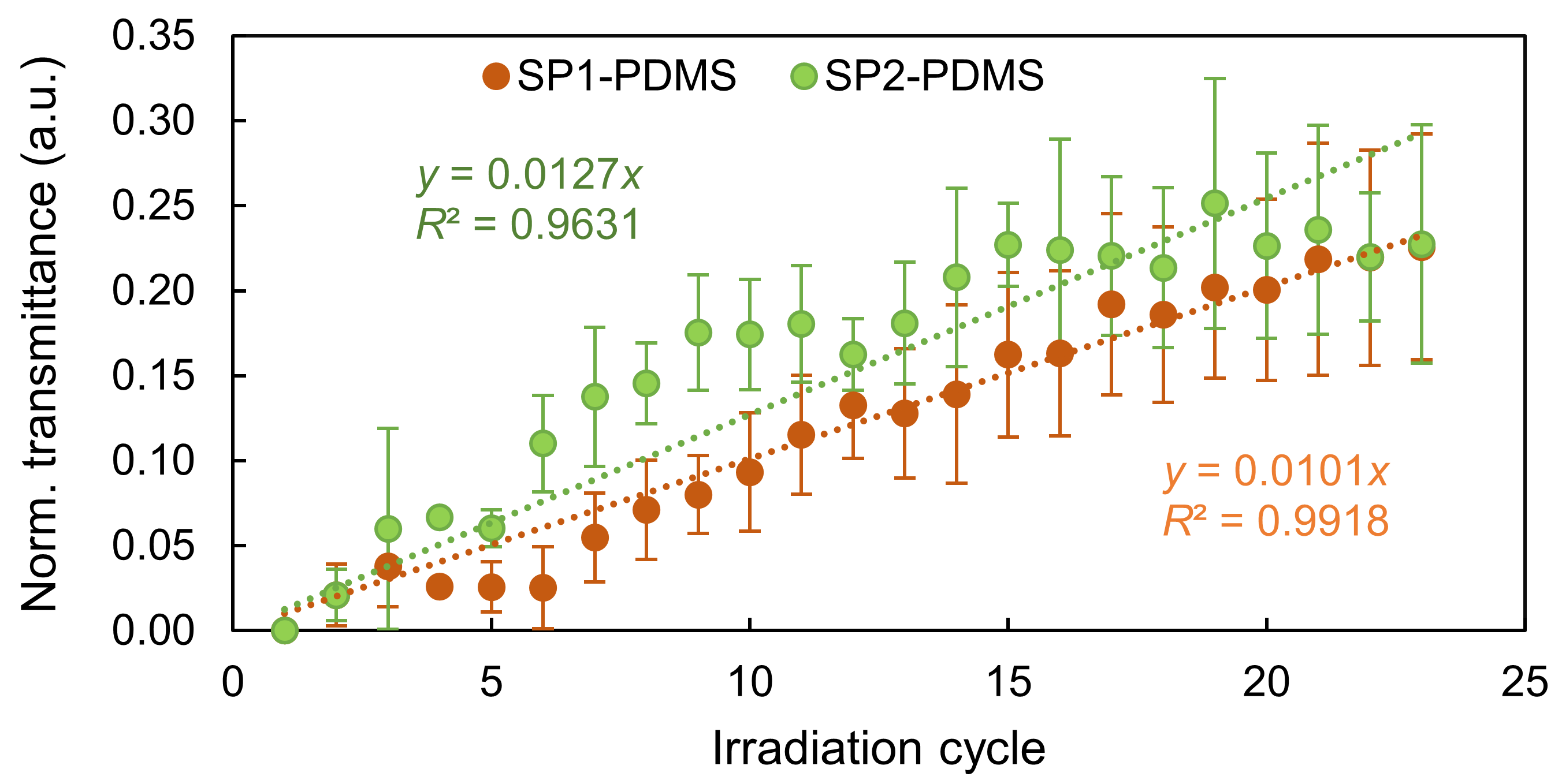 |
| --- |

**Fig. S4** Transmittance evolution of SP1-PDMS and SP2-PDMS free standing films over 23 UV-dark cycles. Error bars indicate the sample standard deviation in independent measurements on three free-standing films

# SP1-PDMS sensing performance

The SP1-PDMS calculated rate (*k*) and time constants (*τ*) for percentage power decay and recovery are given in Table S2, at several testing conditions. Other sensor parameters, i.e., sensitivity, percentage power at saturation, and UV saturation dose, are also presented.

**Table S2** Calculated sensor parameters at different testing conditions

| **Illum.** | **Length**  **(cm)** | ***T***  **(°C)** | **Bend radius**  **(cm)** | ***k_d_* (s^-1^)** | ***τ_d_* (s)** | ***k_r_* (s^-1^)** | ***τ_r_* (s)** | ***S***  **(****%∙cm^2^/J)** | **Saturation point** | |
| --- | --- | --- | --- | --- | --- | --- | --- | --- | --- | --- |
|  |  |  |  |  |  |  |  |  | **∆P/P_0_ (%)** | **UV dose**  **(J/cm^2^)** |
| Dark | 10 | 23.5 ± 1.0 | ∞ | 0.0239  ± 0.0015 | 41.9  ± 2.7 | 0.0094  ± 0.0005 | 107.2  ± 6.0 | 115.5 ± 5.5 | 29.5 ± 3.0 | 0.40 ± 0.02 |
| Dark | 10 | 23.5 ± 1.0 | **0.21** | 0.0237  ± 0.0015 | 42.3  ± 2.6 | 0.0092  ± 0.0006 | 109.0  ± 7.3 | 107.0 ± 0.7 | 27.0 ± 2.3 | 0.37 ± 0.03 |
| Dark | 10 | 23.5 ± 1.0 | **0.34** | 0.0229  ± 0.0029 | 41.4  ± 1.1 | 0.0095  ± 0.0007 | 105.3  ± 7.7 | 114.4 ± 5.0 | 27.3 ± 2.2 | 0.38 ± 0.05 |
| Dark | 10 | 23.5 ± 1.0 | **0.62** | 0.0242  ± 0.0011 | 41.4  ± 1.9 | 0.0097  ± 0.0004 | 103.6  ± 4.4 | 110.5 ± 4.2 | 26.5 ± 3.2 | 0.40 ± 0.05 |
| Dark | 10 | 23.5 ± 1.0 | **1.01** | 0.0241  ± 0.0031 | 41.5  ± 5.3 | 0.0010  ± 0.002 | 101.2  ± 4.1 | 109.5 ± 5.5 | 27.5 ± 5.9 | 0.39 ± 0.05 |
| Dark | **15** | 23.5 ± 1.0 | ∞ | 0.0273  ± 0.0009 | 36.7  ± 1.2 | 0.0080  ± 0.0002 | 124.9  ± 3.6 | 511.8 ± 6.6 | 68.1 ± 0.8 | 0.36 ± 0.01 |
| Dark | **19** | 23.5 ± 1.0 | ∞ | 0.0327  ± 0.0016 | 30.7  ± 1.5 | 0.0067  ± 0.0002 | 148.5  ± 4.4 | 788.4 ± 9.3 | 86.4 ± 0.8 | 0.31 ± 0.01 |
| **White light** | 10 | 24.0 ± 1.3 | ∞ | 0.0217  ± 0.0035 | 48.2  ± 3.1 | 0.0109  ± 0.0005 | 93.6  ± 2.7 | 117.6 ± 6.3 | 34.4 ± 5.0 | 0.56 ± 0.06 |
| White light | 10 | **34.0 ± 0.1** | ∞ | 0.0310  ± 0.0043 | 32.7  ± 4.5 | 0.0205  ± 0.0018 | 48.9  ± 4.0 | 138.2 ± 1.2 | 24.4 ± 6.4 | 0.33 ± 0.03 |
| White light | 10 | **43.5 ± 0.1** | ∞ | 0.0432  ± 0.0026 | 23.2  ± 1.4 | 0.0422  ± 0.00985 | 24.7  ± 5.7 | 149.8 ± 3.3 | 19.2 ± 3.7 | 0.26 ± 0.01 |
| White light | 10 | **52.8 ± 0.2** | ∞ | 0.0642  ± 0.0048 | 15.6  ± 1.1 | 0.0823  ± 0.0198 | 12.7  ± 3.2 | 157.0 ± 5.7 | 12.9 ± 2.1 | 0.22 ± 0.03 |
| White light | 10 | **61.6 ± 0.2** | ∞ | 0.0919  ± 0.0099 | 11.0  ± 1.1 | 0.1477  ± 0.0195 | 6.9  ± 1.0 | 174.9 ± 3.5 | 7.5 ± 1.9 | 0.19 ± 0.02 |
| White light | 10 | **70.5 ± 0.3** | ∞ | 0.1515  ± 0.0090 | 6.6  ± 0.4 | 0.1510  ± 0.0192 | 6.7  ± 0.8 | 187.3 ± 0.9 | 4.3 ± 1.0 | 0.11 ± 0.03 |

## Bending effect

A representative plot of neat PDMS and SP1-PDMS responses to UV light at various bend radii is shown in Fig. S5a. SP1-PDMS and neat PDMS normalized transmitted optical power as a function of bend radii and optical losses in dB as a function of curvature are presented in Fig. S5b–c, respectively. The transmitted optical power of the straight waveguides was taken as a reference for the normalization shown in Fig. S5b.

| **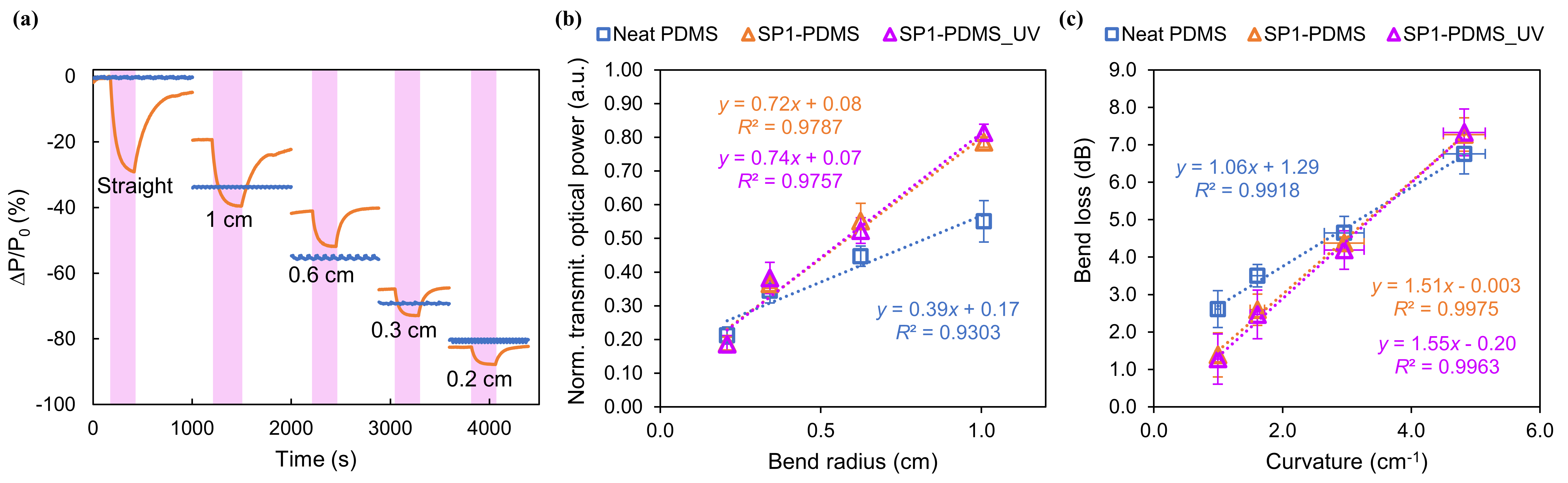** |
| --- |

**Fig. S5 a** Representative plot of neat PDMS and SP1-PDMS responses to UV light (pink shaded area) with time at various bend radii. **b** Normalized transmitted optical power as a function of bend radii, and **c** optical power losses at 633 nm at various curvatures and their respective best-fit lines. Error bars indicate the sample standard deviation in independent measurements on three waveguides

## Effect of length

A representative plot showing the SP1-PDMS response to three cycles of UV light at different waveguide lengths is depicted in Fig. S6a. The calculated sensor parameters given in Table S2 were plotted as a function of waveguide length and fitted by linear regression, as shown in Fig. S6b–e.

| **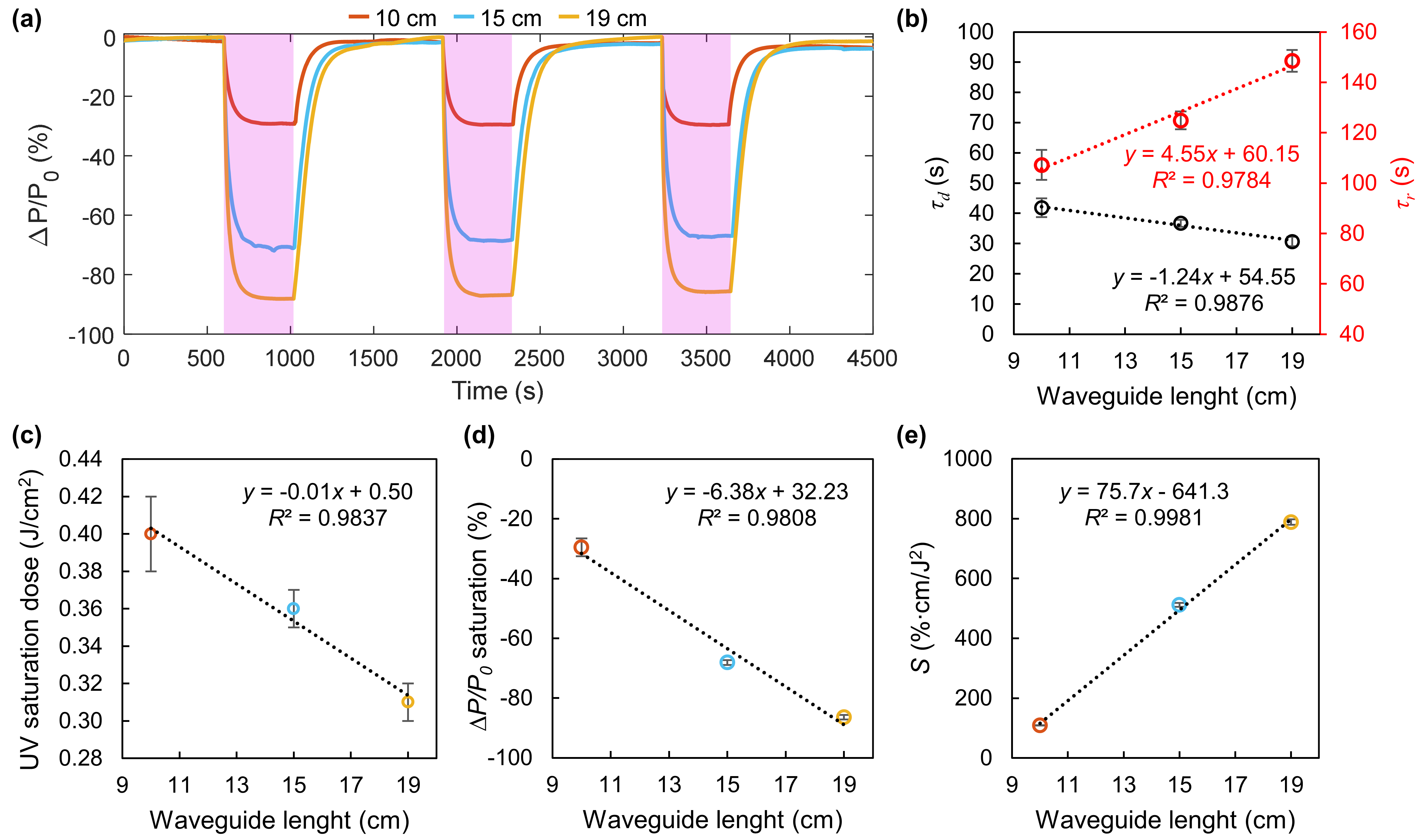** |
| --- |

**Fig. S6 a** Representative SP1-PDMS response change with waveguide length. **b** Decay and recovery time constants, **c** UV saturation dose, **d** percentage power change at saturation, and **e** sensitivity as a function of waveguide length. Error bars indicate the sample standard deviation in independent measurements on three waveguides

The decay time constant and UV saturation dose decreased as the waveguide length increased, allowing it to reach photostationary state (saturation) faster due to increased optical losses along the propagation length. It also resulted in longer recovery time constants.

# References

1. Tian, W., Tian, J.: An insight into the solvent effect on photo-, solvato-chromism of spiropyran through the perspective of intermolecular interactions. Dyes Pigments. 105, 66–74 (2014). https://doi.org/10.1016/j.dyepig.2014.01.020

2. Kortekaas, L., Browne, W.R.: The evolution of spiropyran: fundamentals and progress of an extraordinarily versatile photochrome. Chem. Soc. Rev. 48, 3406–3424 (2019). https://doi.org/10.1039/C9CS00203K

3. Bicerano, J.: Prediction of Polymer Properties. Marcel Dekker, New York, USA. (2002)

4. Aranguren, M.I.: Crystallization of polydimethylsiloxane: effect of silica filler and curing. Polymer. 39, 4897–4903 (1998). https://doi.org/10.1016/S0032-3861(97)10252-X

5. Allen, F.H., Wood, P.A., Galek, P.T.A.: Role of chloroform and dichloromethane solvent molecules in crystal packing: An interaction propensity study. Acta Crystallogr. Sect. B Struct. Sci. Cryst. Eng. Mater. 69, 379–388 (2013). https://doi.org/10.1107/S2052519213015078

6. Klajn, R.: Spiropyran-based dynamic materials. Chem. Soc. Rev. 43, 148–184 (2014). https://doi.org/10.1039/c3cs60181a

7. Minkin, V.I.: Photo-, thermo-, solvato-, and electrochromic spiroheterocyclic compounds. Chem. Rev. 104, 2751–2776 (2004). https://doi.org/10.1021/cr020088u

8. Lin, J.-S.: Interaction between dispersed photochromic compound and polymer matrix. Eur. Polym. J. 39, 1693–1700 (2003). https://doi.org/10.1016/S0014-3057(03)00058-2
